# Linearly acenaphthylene-fused pentacene exhibiting efficient singlet fission

**DOI:** 10.1039/d5sc08246c

**Published:** 2026-06-02

**Authors:** Jacob Arvidson, Saad Shaikh, Masahiro Tanaka, Tejal Pawale, Andrew Dawson, Xiao Li, Vladimir N. Nesterov, Yasuhiro Kobori, Somnath Das, Hong Wang

**Affiliations:** a Department of Chemistry, University of North Texas Denton TX 76203 USA hong.wang@unt.edu dassom08@gmail.com; b Materials Science and Engineering Department, University of North Texas 3940 North Elm Str Denton Texas 76209 USA; c Department of Chemistry, Graduate School of Science, Kobe University 1-1 Rokkodaicho Nada-ku Kobe 657-8501 Japan ykobori@kitty.kobe-u.ac.jp; d Laser Molecular Photoscience Laboratory, Molecular Photoscience Research Center, Kobe University 1-1 Rokkodaicho Nada-ku Kobe 657-8501 Japan

## Abstract

Acenes are poised to be highly sought after for singlet fission, which is among the most promising strategies for scaling solar power conversion efficiency beyond the Shockley–Queisser limit. One critical problem associated with acenes is their low stability. Incorporation of a 5-membered ring into acenes is reported to stabilize acenes. However, cyclopentannulation on acenes at the *peri*-positions also transforms the electronic structure of acenes and endows them with nontypical photophysical properties, thus preventing them from undergoing singlet fission. Here, we report a new strategy to stabilize and simultaneously retain the photophysical properties of the acenes through linearly fusing acenaphthylene containing a 5-membered ring. The acenaphthylene-fused pentacenes exhibited UV-Vis absorption and fluorescence spectra with distinctively red-shifted absorption and emission bands, notably along with a substantially longer fluorescence lifetime, while still retaining the characteristic spectral features of pentacene. Despite possessing narrower HOMO–LUMO energy gaps and elevated HOMO energy levels, the elongated acenes exhibited stability comparable to that of their pentacene analogues. X-ray crystallography revealed a slip-stack columnar array in these molecules, a packing motif that differs significantly from the herringbone packing typically seen in pentacene. Strong vibronic coupling—the interrelation of electronic and vibrational motions—and favorable excited-state energetics drive an efficient, ultrafast singlet fission in thin films, resulting in a high triplet quantum yield of ∼158%. TREPR spectroscopy confirmed the singlet fission mechanism—specifically the formation of triplet pairs—by resolving the sequence of spin-state changes and revealing the elusive quintet (^5^TT) state.

## Abstract 2

Fusing acenaphthylene to pentacene is proven to be an effective strategy to stabilize pentacene. The acenaphthylene-fused pentacene underwent efficient ultrafast singlet fission in thin films, achieving a triplet quantum yield of ∼158%.

## Graphical abstract

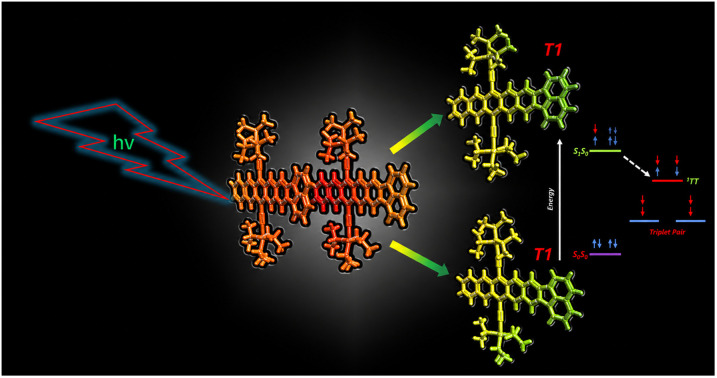

## Introduction

**Fig. 1:**
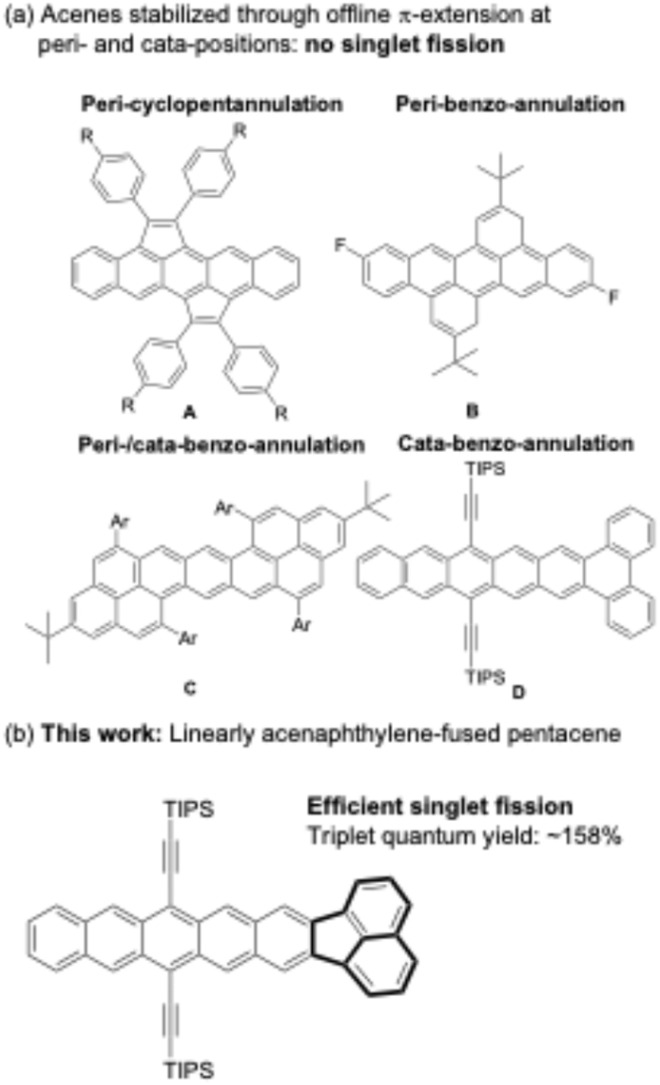
(a) Selected examples of acenes stabilized through offline π-extension. (b) This work.

Acenes are a unique class of polycyclic aromatic hydrocarbons (PAHs) composed of linearly fused benzene rings. Organic electronic materials based on acenes show promise to make revolutionary transformations in a range of applications^1–10^ such as photovoltaics, organic field-effect transistors (OFETs), and organic light-emitting devices (OLEDs) due to their exceptional charge mobility. In photovoltaics, acenes are considered benchmark compounds for singlet fission,^11–16^ a spin-conserving phenomenon to distribute one high-energy singlet exciton into a pair of two (or even more) times lower-energy triplets, thereby effectively circumventing the Shockley–Queisser limit (∼33.7%) in solar cells.^17–22^ One unique and interesting feature of acenes is that their charge mobility increases exponentially with the increase in their length. As such, it has remained a hot research topic to obtain higher acenes.^2,5,15,23–38^ However, a critical problem associated with higher acenes is their low stability. As the length of acenes increases, the stability drops quickly due to much more facile oxidative decomposition arising from the quickly shrinking HOMO–LUMO gap. Significant research effort has focused on this area, and strategies such as increasing steric bulkiness and substitution with strongly electron-withdrawing groups have been developed to stabilize acenes.^23,24,26,27,29,39–52^ Extremely sterically hindered acenes,^53–56^*i.e.*, twisted acenes and heterocenes,^57–61^ where one or more carbons of the acenes are replaced by heteroatoms, have also been introduced to address the stability issue. In recent years, offline π-extension, including cyclopentannulation (A)^62–70^ and benzo-annulation^71–74^ (B, C, and D), has emerged as an effective strategy to stabilize acenes (Fig. 1a). Five-membered rings have non-alternant electronic structures and can serve as electron acceptors.^62,64–66^ Due to these features, incorporation of a 5-membered ring into acenes is expected to not only further stabilize the electron-rich acenes but also bring in unprecedented novel properties. However, almost all reported cyclopentannulation on acenes is at the *peri*-positions,^62–70^ which gives rise to electronic and photophysical properties non-typical of acenes. As a result, singlet fission has not been reported for cyclopentannulated acenes. In this work, we introduce a new approach to incorporate a five-membered ring into acenes through linear fusion of acenaphthylene to pentacene (Fig. 1b). The acenaphthylene-fused pentacenes, featuring more linearly *cata*-fused rings, showed stability comparable to or better than their pentacene analogues. Crucially, they maintained their optical and photophysical properties, achieving an efficient, ultrafast singlet fission with a triplet quantum yield of approximately 158% in thin films.

Acenaphthylene is composed of a naphthalene and an ethylene bridge located at a fused 5-membered ring. Despite having 12 π-electrons, acenaphthylene is overall aromatic with the five-membered ring showing only slight anti-aromaticity.^75–78^ Our goal is two-fold: first, we investigate the impact of acenaphthylene on the stability of the resulting molecular system. Second, we investigate whether the resulting molecular system retains the photophysical properties of acene necessary to facilitate singlet fission. In addition, the incorporation of an acenaphthylene unit into pentacene will introduce asymmetry into the resulting acenes, a factor that has rarely been investigated for crystal packing. Herein, the impact of the acenaphthylene unit on the optical properties, stability, crystal packing, and ultrafast processes of a linearly modified acene (Ace-PCSi) was investigated using steady-state and femtosecond time-resolved transient absorption spectroscopies and X-ray crystallography. Our studies have unveiled new photoinduced ultrafast carrier dynamics involving underlying triplet states in Ace-PCSi for singlet fission. Time-resolved electron paramagnetic resonance (TREPR) spectroscopy was used to elucidate the spin-state evolution during the occurrence of singlet fission. Favourable energetics of the associated excited-states and strong intermolecular vibronic interactions facilitating such efficient singlet-triplet evolutions have also been tracked synergically through DFT calculations and X-ray crystallography of the new acene.

## Results and discussion

### Design and synthesis of acenaphthylene-fused pentacene

**Scheme 1:**
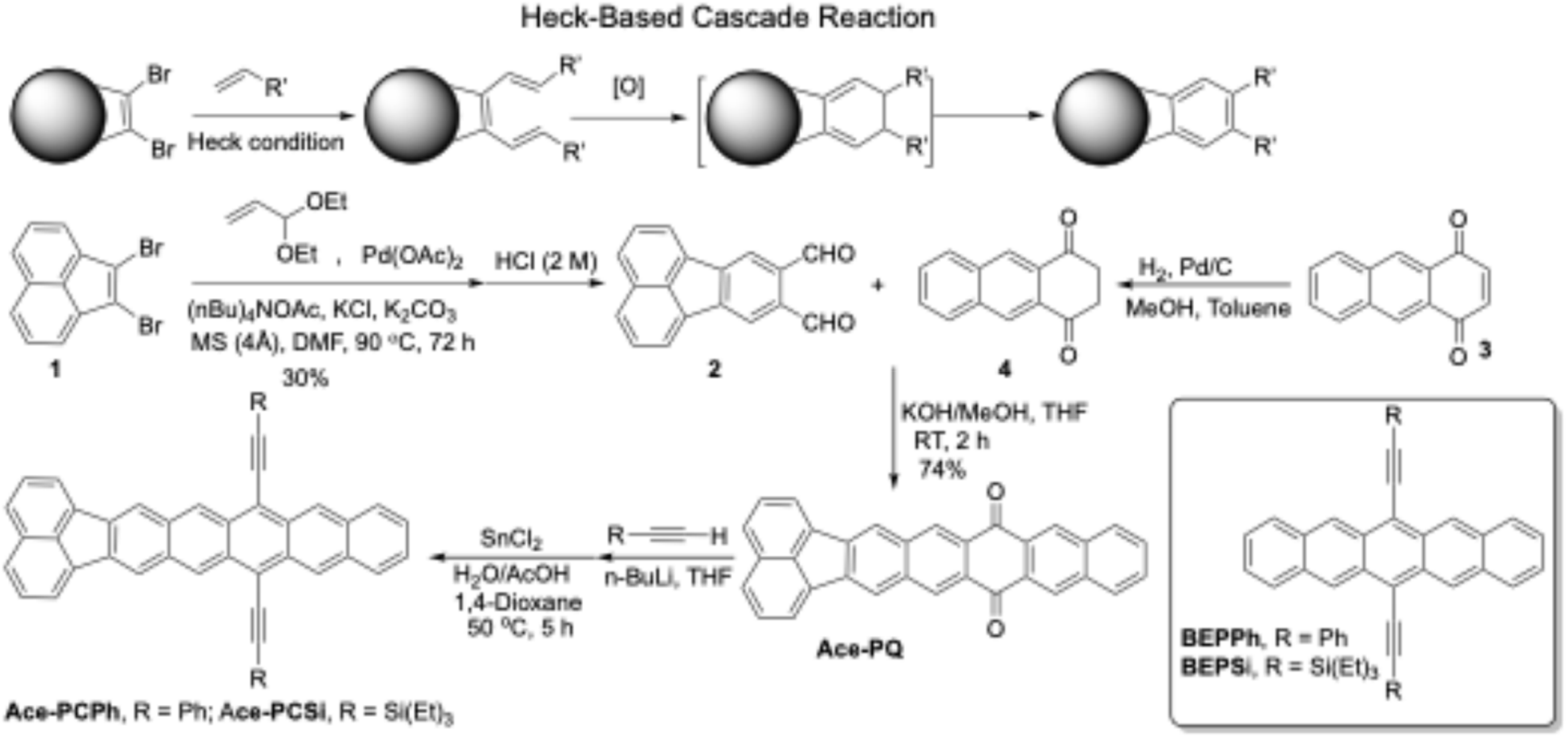
Synthesis of the acenaphthylene-fused pentacenes Ace-PCPh and Ace-PCSi.

To synthesize a linearly acenaphthylene extended acene, we planned to first prepare fluoranthene-8,9-dicarbaldehyde 2. Fluoranthene-8,9-dicarbaldehyde 2 has not been reported in the literature, and therefore, a synthetic method must be developed to access 2. We decided to attempt a Heck reaction-based cascade reaction on 1,2-dibromoacenaphthylene 1 (Scheme 1), which was developed in our laboratory to access functionalized benzoporphyrins.^79^ Acenaphthylene is a useful building block for π-electron functional materials.^77,78^ The development of a new synthetic strategy to functionalize acenaphthylene will open new doors for functional materials.

The Heck reaction-based cascade reaction, which involves a Heck reaction, a 6 π-cyclization, and aromatization (Scheme 1), appears to be an ideal approach to serve this purpose. The Heck-based cascade reaction was first attempted with acrolein and 1,2-dibromoacenaphthylene 1 (ref. 80) through optimizing established conditions. However, a complex mixture always resulted, likely due to the unstable nature of both 1 and acrolein. Fortunately, the Heck-based cascade reaction was successfully carried out under colloidal Heck conditions^81,82^ with acrolein diethyl acetal as the alkene. Under the colloidal Heck conditions, tetrabutylammonium acetate was used as a phase transfer reagent, potassium chloride as an inorganic additive to assist in the colloid formation, potassium carbonate as a base, and palladium acetate as the Heck catalyst. It was observed that when water was present, total oxidation would not occur, and the unoxidized fluoranthene-8,9-dicarbaldehyde was the major product. The addition of activated 4 Å molecular sieves removed water from the solution and facilitated the reaction. After 72 hours, the reaction crude was hydrolyzed with diluted HCl to generate the desired fluoranthene-8,9-dicarbaldehyde 2 in a single step with 30% yield.

Fluoranthene-8,9-dicarbaldehyde 2 was treated with 2,3-dihydroanthracene-1,4-dione 4, which was obtained through the reduction of commercially available anthracene-1,4-dione 3, under basic conditions through double aldol condensation to give Ace-PQ. It is notable that 2,3-dihydroanthracene-1,4-dione 4 is highly sensitive to air; the reduction and the subsequent aldol condensation reactions were carried out under air-free conditions. Despite Ace-PQ being completely insoluble in all common solvents, Ace-PQ smoothly underwent an addition reaction with acetylide, followed by reductive aromatization using stannous chloride and acetic acid to generate the acenaphthylene-fused pentacenes Ace-PCPh and Ace-PCSi. For comparison purposes, pentacenes BEPPh and BEPSi were also prepared using a similar approach. Ace-PCPh was originally prepared first. However, attempts to prepare thin films using Ace-PCPh failed due to its low solubility. We then switched to the 6,13-bis(triisopropylsilylethynyl) (TIPS) substituted Ace-PCSi, as the TIPS-substituents provide better solubility. Thin films needed for the singlet fission study were successfully obtained using this derivative.

### Optical properties and DFT calculations

**Fig. 2:**
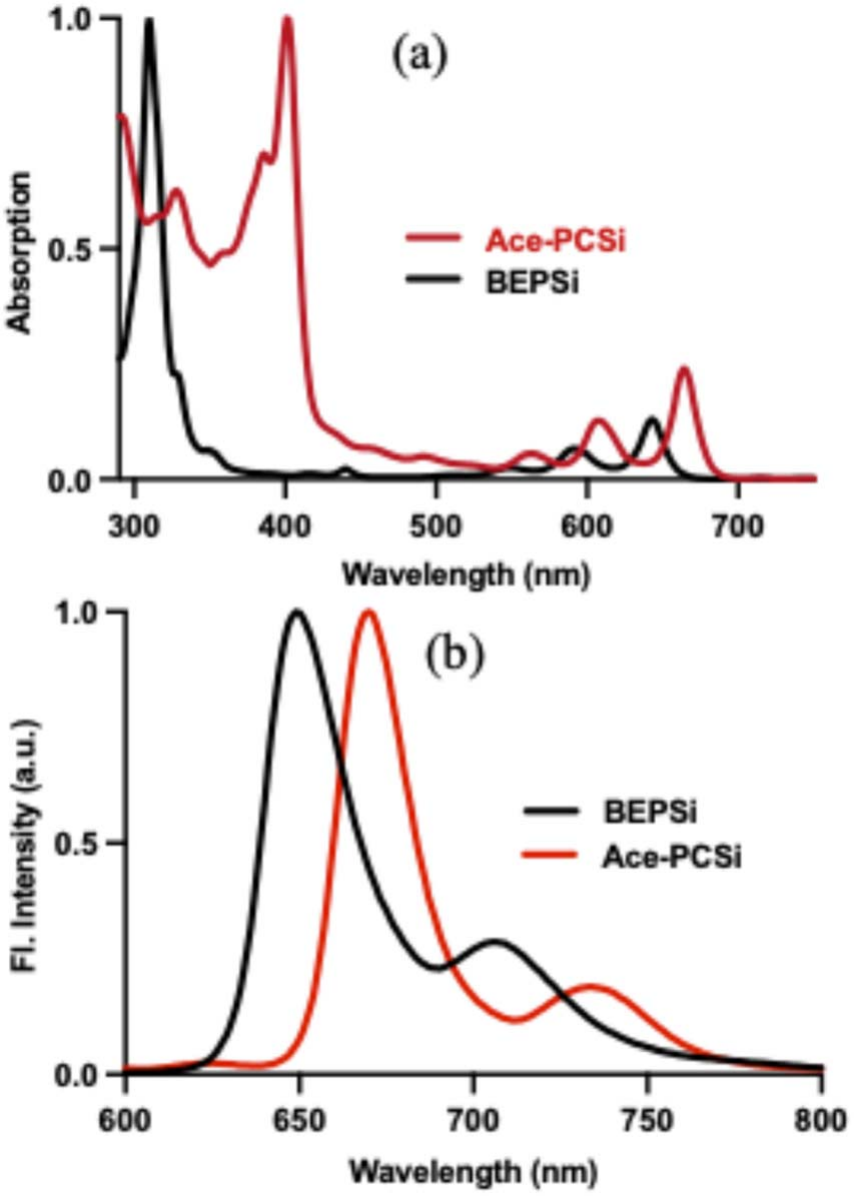
Normalized (a) UV-Vis absorption spectra and (b) fluorescence spectra of Ace-PCSi and BEPSi in toluene. Excitation at 457 nm for Ace-PCSi and 440 nm for BEPSi.

The optical properties of Ace-PCPh and Ace-PCSi were measured by UV-Vis absorption and fluorescence spectroscopies (Fig. 2, S1 and S2). Unlike the *peri*- and *peri*-/*cata*-annulated acenes (Fig. 1, A, B, and C),^62–67^ the UV-Vis absorption spectra of both Ace-PCPh and Ace-PCSi displayed typical characteristic α, β and p bands of acenes, which were found at 402, 496, and 678 nm, and 401, 493, and 664 nm, respectively. It is notable that the β bands of Ace-PCPh and Ace-PCSi showed the largest bathochromic shifts relative to those of their pentacene analogues by ∼45 and 92 nm, respectively. The α and p bands were also significantly red-shifted, highlighting the effect of π-extension. Consistent with the absorption results, the emission bands of Ace-PCSi are red-shifted compared to those of BEPSi. Notably, considering the lowest energy absorption and highest energy emission peaks at ∼664 and 678 nm, respectively, the singlet state (S_1_) energy for Ace-PCSi could be calculated as ∼1.86 eV. Its triplet state (T_1_) appears at ∼0.80 eV based on phosphorescence data (Fig. S3 in the SI). Interestingly, more than twice the energy for the S_1_ over T_1_ state indicates a bright prospect of Ace-PCSi towards efficient singlet fission.^51,52^ Both the HOMOs and LUMOs of Ace-PCSi and Ace-PCPh from DFT calculations (Fig. S12 and S13 in the SI) are centered at the pentacene unit with only slight involvement of the acenaphthylene unit, which explains their characteristic absorption bands in the UV-Vis absorption spectra. The HOMO shows slightly greater participation of the acenaphthylene unit than the LUMO. The HOMO energy level of Ace-PCSi is elevated compared to that of BEPSi, and the LUMO is slightly lowered (Fig. S14 in the SI). As a result, the calculated HOMO–LUMO energy gap of Ace-PCSi (1.90 eV) is narrower than that of pentacene BEPSi (1.93 eV), which agrees with the red-shifted vibronically structured (finger structure) UV-Vis spectra of Ace-PCSi and the optical band gaps (1.80 eV and 1.85 eV for Ace-PCSi and BEPSi, respectively, Table S1 in the SI). On the other hand, the acenaphthylene unit heavily contributes to the HOMO−1 and LUMO+1 of Ace-PCSi and Ace-PCPh. The energy level of the LUMO+1 is significantly lowered, and that of the HOMO−1 is significantly elevated compared to those of BEPSi and BEPPh, which is reflected by the largely red-shifted β bands in the UV-Vis spectra of Ace-PCSi and Ace-PCPh. The fluorescence spectra of Ace-PCSi displayed two emission bands at 670 nm and 734 nm, which are significantly red-shifted relative to those of BEPSi, consistent with the steady-state absorption and computational data. It is notable that Ace-PCSi has a longer fluorescence lifetime than BEPSi, with measurements of 15.3 ns (in air) and 20.4 ns (in argon), *versus* 12.6 ns and 17.3 ns, respectively (Fig. S11 in the SI). To further understand the electronic transitions, we used TD-DFT calculations to assign the absorption bands of BEP-Si and Ace-PCSi (Fig. S4 and S5 in the SI).

The photostability of Ace-PCPh, BEPPh, Ace-PCSi, and BEPSi was monitored by UV-Vis absorption spectroscopy in 0.01 mM benzene solution under constant exposure to light and air (Fig. S6–S10 in the SI). The half-life was determined to be 45 h (Ace-PCPh), 33 h (BEPPh), 688 min (Ace-PCSi), and 795 min (BEPSi). Overall, the acenaphthylene-fused pentacenes exhibited superior or comparable stability to their pentacene analogues. Ace-PCPh and Ace-PCSi are linearly π-extended and can be deemed as longer acenes than pentacene. The stability results are surprising given the higher HOMO energy levels and narrower HOMO–LUMO gaps, which typically suggest lower stability. The data in this section highlight how the addition of an acenaphthylene unit perturbs the electronic characteristics of acenes.

### Single crystal X-ray crystallography

**Fig. 3:**
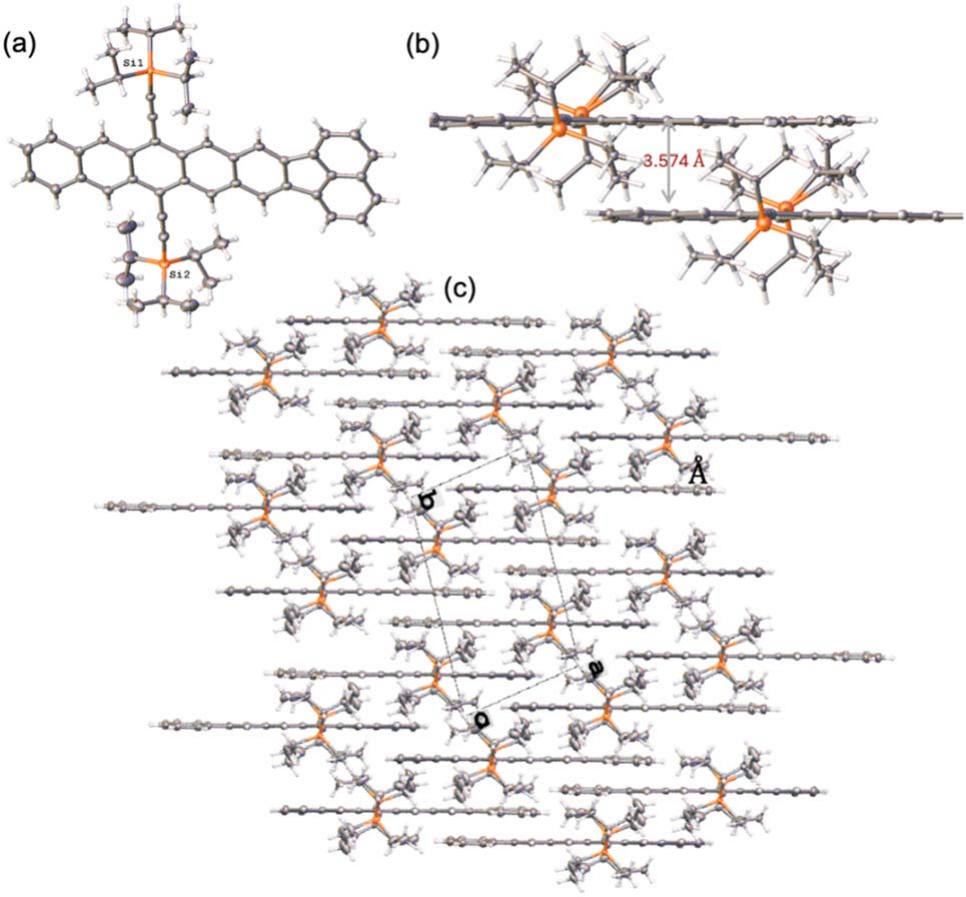
Ace-PCSi: (a) X-ray crystal structure, (b) crystal packing mode of two adjacent molecules, (c) a packing motif.

Single crystal structures of Ace-PCSi and BEPPh were resolved (Fig. 3 and S32–S34 in the SI, CCDC 2450036 and 2450035, respectively). Ace-PCSi adopts a strictly planar yet asymmetrical geometry. Notably, an asymmetric pentacene reported by the Anthony group adopts a herringbone packing.^83^ In sharp contrast, the Ace-PCSi assumes a brick wall packing pattern with significant overlap of the adjacent molecules (Fig. 3b), highlighting the effect of the acenaphthylene. This is intriguing considering the asymmetric structure of Ace-PCSi. The herringbone packing results in different layered structures with both face-to-edge and edge-to-edge modes. The brick wall packing pattern is expected to facilitate long-range intermolecular interactions through maintaining substantial face-to-face overlap. The interplanar distance between two closest Ace-PCSi molecules is 3.574 Å. Notably, the single-crystal structure of BEPPh was also obtained, revealing a herringbone packing motif similar to that of unsubstituted pentacene.^84–88^

### Transient absorption spectroscopy

**Fig. 4:**
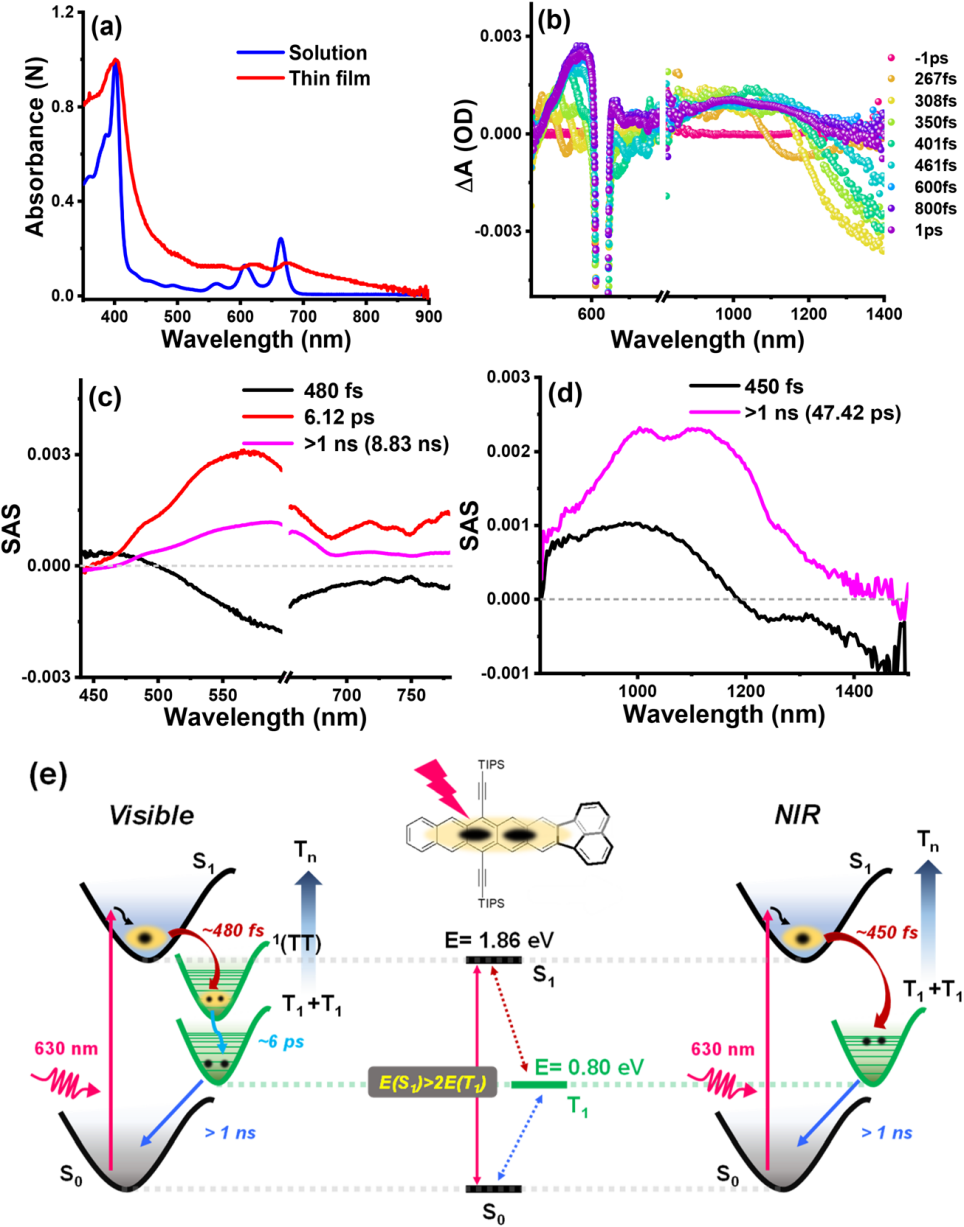
(a) Ground state absorption feature of Ace-PCSi in toluene and spin-coated thin films. (b) fs-TA spectral evolutions at the indicated probe delays in the early time scale. (c and d) SAS was achieved after the GTA of the fs-TA data in the visible and NIR windows, respectively. The SAS representing 47.42 ps ideally possesses a time constant of 3.91 ns (>1 ns) upon complete fitting to the offset. (e) A schematic representation of the excited-state dynamics and energetics of different states involved in the singlet fission of Ace-PCSi. For simplicity, the potential energy surface of one T_1_ state is shown after the first step of singlet fission.

The favorable packing patterns with closer π-stacked systems in the solid-state indicate that there could be a strong intermolecular electronic coupling between two adjacent Ace-PCSi molecules, which is clearly reflected from its red-shifted and relatively broad vibrational progression absorption bands in the thin film (Fig. 4a). In contrast to the solution, the broad and red-shifted vibrational bands in the solid state suggest a J-aggregate-type spectrum^89,90^ of Ace-PCSi with strong intermolecular vibronic coupling, which has also been confirmed with Powder X-Ray Diffractometry (PXRD) conducted for the thin film (Fig. S35 in the SI). Additionally, excited-state DFT calculations also provide excited-state energies of the S_1_ and T_1_ states at 1.90 and 0.63 eV, respectively, demonstrating *E*(S_1_) > 2*E*(T_1_) and thus satisfying the basic energy requirements for singlet fission (Fig. S19 in the SI).^91–94^ Prompted by these initial prerequisites, we then tried to disentangle the interplay of excited-state events as photoinduced singlet fission using ultrafast transient absorption (TA) spectroscopy in thin films as they closely replicate the operational conditions (*i.e.* solid-state environment) under which solar energy absorbers function in contemporary light-conversion applications. The TA spectroscopic data were specifically collected from the Ace-PCSi thin films (thickness of ∼10 nm) using 630 nm excitation. The thickness and morphology of the films were measured with AFM/ellipsometry and PXRD, respectively (Fig. S36 and S37 in the SI). The efficiency of singlet fission greatly depends on competitive parasitic non-/radiative deactivations with effective generation of correlated triplet pairs in the ultrafast time domain,^93^ and Fig. 4b summarizes the early-time (up to ∼1 ps) spectral behavior of the Ace-PCSi thin film in the visible and near infrared (NIR) window. Significant spectral evolution is observed in the early stages of both regions. For example, the excited state absorption (ESA) in the visible exhibits continuously evolving signals at ∼470–500 nm before ∼500 fs and finally peaks at ∼550–560 nm around 0.8–1 ps (Fig. 4b). A similar spectral signature in initial delay for TIPS-pentacene was attributed to the ESA feature of states with different multiplicities by Ramanan *et al.*, *vis-à-vis* singlet fission mediated S_1_ to T_1_ conversion.^95^ The early time short wavelength (<500 nm) spectral features thus can be assigned to ESA related to singlets (S_1_ → S_*n*_), which subsequently undergo an ultrafast conversion into triplets, leading to triplet ESA (T_1_ → T_*n*_) at ∼550–560 nm. Such an ultrafast transition of singlets into triplets is not surprising, as efficient singlet fission is believed to proceed *via* a spin-conserved (and thus faster) intermediate, known as correlated triplet pair ^1^(TT).^96–99^ Similarly, in the NIR, a relatively narrow ESA appearing at ∼850–1100 nm before ∼500 fs quickly evolves into a broader absorption band by <1 ps, covering almost the entire detectable NIR window (Fig. 4b). These synergistically evolved ultrafast spectral transitions in the NIR are also consistent with those observed in the visible and are thus attributed to the characteristic conversion of the directly formed singlets into triplets following photoexcitation.^100–102^ Although a comparison of similar experiments done in dilute solution and compact films is trivial, the relative position of ESAs obtained upon 630 nm photoexcitation of 0.01 mM Ace-PCSi in toluene at ∼475 and 850–1100 nm, respectively, in the visible and NIR related to singlet states, is found to be closely comparable to that observed in thin film of the compound (Fig. S20 in the SI). Note that poor intermolecular interaction between Ace-PCSi molecules in such a dilute medium produces spectral features predominantly of the singlet states even after >2 ns, in stark contrast to those of the solid thin films. Consistent with this, rubrene populates its singlet excited state in solution predominantly due to inefficient intersystem crossing (a radiationless unimolecular process that competes with singlet fission), whereas in the solid single-crystalline state, triplet absorption arising from efficient singlet fission dominates.^116^ These observations underscore the decisive role of intermolecular electronic coupling in enabling exothermic singlet fission in the solid state of polyaromatic hydrocarbons. The intermediate ^1^(TT) state formed latest by 1 ps upon photoexcitation subsequently undergoes spectrally silent relaxation into independent triplets (T_1_), and thus, no new spectral signature appears in a longer time window for the triplets before deactivation to the ground state (S_0_) (Fig. S21 in the SI).

Insight into the spectral features of the involved species from the overlapping TA signals, along with their associated time constants, was further obtained by performing global target analysis (GTA) of the visible and NIR datasets independently; the results are summarized in Fig. 4c and d, respectively. Based on previous reports on pentacene derivatives, a sequential relaxation model has been introduced on GTA to deconvolute spectral information of the photoexcited processes in the Ace-PCSi thin film.^100–103^ The first species associated spectrum (SAS) in the visible appears as a broad spectrum with significant contribution in the bluer side (∼450–490 nm) of the region with no-to-negative contribution after ∼500 nm, indicating that the S_1_ state forms directly after photoexcitation and decays with a time constant of ∼480 fs (Fig. 4c). It is notable that the time constant for S_1_ state decay of Ace-PCSi is significantly longer than that reported for pentacene and its derivatives (50–200 fs).^100,103^ Although acenaphthylene is overall aromatic, the five-membered ring possesses slight anti-aromaticity. We suspect that upon excitation, it becomes aromatic in the excited state according to Baird's rule, and thus, a more stable Sπ state with a prolonged lifetime was observed.^104–108^ However, NICS analysis reveals slight antiaromaticity of the 5-membered ring and decreased aromaticity of the adjacent benzene ring in ^0^S, ^1^S and ^1^T states (Table S2 in the SI). The blue end of the other two species is much less pronounced though. Instead, a stronger and more intense ESA feature associated with ^1^(TT) (red) emerges, centered after 550 nm, with a lifetime of approximately 6.12 ps. It is important to note that the involvement of two different types of interconvertible correlated triplet-pair intermediates (spatially interacting and noninteracting) on timescales of a few ps had been previously proposed during singlet fission of pentacene derivatives.^97,99,103^ Triplet energy transfer, leading to a change in excitonic interactions and occurring on a ps time frame, was reported to be key in facilitating such interconversion of crystalline acenes.^99,109,110^ Notably, in our case, the ∼6.12 ps time constant associated with this second, *i.e.*, correlated ^1^(TT) state, is closely comparable with the triplet energy transfer rate in crystalline pentacenes, and therefore participation of two spectrally identical and spatially interconvertible triplet pair intermediates can't be ruled out. The third SAS, representing the independent T_1_ states with similar spectral features to those of correlated triplet pairs, finally decays to the ground S_0_ state with a time constant of >1 ns.^98,100–102^

Unlike visible, the assessment of S_1_/T_1_ spectral characteristics in the NIR for pentacene (and its derivatives) has historically been counterintuitive, with some reports claiming to be free of any S_1_ spectral contribution,^111,112^ while others demonstrated an overlapping S_1_ → T_1_ signal in this region.^100,113^ However, here the calculated sub-unity energy for the T_1_ state promises to exhibit signature absorption in the NIR due to the transition between triplet energy states of Ace-PCSi. That being said, owing to the ultrafast early time events of S_1_ to correlated triplet pair formation in the visible, GTA in the NIR has been carried out, taking the initial (up to 50 ps) spectral evolutions into account so that minute spectroscopic information for any such transitions in a shorter time domain is not overlooked. Interestingly, a two-component GTA fitting in the NIR satisfactorily describes the excited-state photoevents in Ace-PCSi (Fig. 4d), whereas a three-component fitting, even over a longer time window (like that in the visible), does not substantially improve the NIR fitting quality (Fig. S22 in the SI). This indicates that photoinduced processes, particularly in the early delay in the NIR, are presumably faster than those in the visible region. This is not surprising as the time constant associated with triplet pair formation in acenes is usually in the order of a few hundred fs and becomes relatively faster with a gradual red-shift (visible-to-NIR) of excitonic singlet absorption owing to modulation in the driving force of this step,^101,102^ and also undergoes a different relaxation pathway in the NIR.^100^ The first component, exhibiting a broad spectrum up to ∼1180 nm, decays with a time constant of ∼450 fs (comparable to that in the visible) and quickly evolves into an even broader spectrum with a lifetime of ∼47.42 ps, spanning nearly the entire NIR window, corresponding, respectively, to the S_1_ and T_1_ states of Ace-PCSi. Notably, since GTA is performed using only a limited initial time window, the second component representing T_1_ ideally possesses a time constant of >1 ns upon complete fitting to the offset; however, a value of ∼3.91 ns is obtained. Needless to mention, this longer decay time for T_1_ before spin-forbidden recombination to the ground state is particularly desirable, as this long-excited state provides a larger time window to effectively extract the photogenerated charge carriers for solar energy conversions. Interestingly, the amplitudes of the deconvoluted S_1_ signals are found to be much less than those of the T_1_ states in both the NIR and visible regions, indicating that transients undergoing triplet absorption are more than the initially populated singlets and thereby suggesting that the linearly cyclopentannulated acenes Ace-PCSi can indeed undergo an efficient singlet fission mediated multiexciton formation in the ultrafast time regime, a phenomenon that has not been seen in this class of acenes. Consequently, a pronounced singlet fission is also exemplified by a calculated triplet yield of ∼158% (see details in the SI), in line with the spectral data, and indicates a lesser extent of possible energy-wasting competitive processes. However, although singlet fission is efficient and occurs on a sub-ps timescale, a 158% triplet yield suggests the presence of unavoidable deactivation processes. Radiative (fluorescence) and non-radiative events from S_1_ are expected to be operative at early times in addition to other deactivation pathways (to S_0_) originating from the intermediate and/or final excited states, particularly as time elapses. However, these have historically been difficult to trace for pentacene derivatives.^103^ Importantly, triplet–triplet annihilation mediated deactivation is unlikely here due to *E*(T_2_) > 2*E*(T_1_) of Ace-PCSi accessed from excited state DFT studies (T_1_ = 0.63 and T_2_ = 2.81 eV). Although Ace-PCSi possesses S_1_/T_1_ spectral signatures in the NIR, a clear distinction between the two states, both in terms of spectral and kinetic information, is evident, particularly after the GTA fittings.^100–102^ Notably, a relatively faster decay of the S_1_ state for the formation of the triplet in the NIR relative to the visible suggests that two different ultrafast channels are associated with the ESAs from T_1_ in the visible and NIR, and thus likely probe different transitions involving different initial vibrational levels on the excited T_1_ potential energy surface (Fig. 4e). For example, while the longer formation time of the correlated triplet pair (*i.e.* decay of S_1_) corresponds to the association of deep (lower) vibrational levels on the triplet potential surface in the visible, the same in the NIR presumably originates from shallow (higher) vibrational levels (Fig. 4e). Furthermore, the absence of a reliable intermediate state with a lifetime of a few ps in the NIR indicates that higher lying triplets (T_*n*_) decay to the ground state of Ace-PCSi following different pathways in these two spectral regions.^100–102^ It is, however, expected that, due to the involvement of underlying triplet states in Ace-PCSi following singlet fission, magnetic-field-dependent photoinduced ultrafast carrier dynamics may open a new avenue in elucidating uncharacterized intermediates and their spectroscopic fingerprints. This work, however, specifically unveils that linearly fused *cata*-π-extended pentacenes can indeed be a solution over *peri*-annulated chromophores to not only improve the overall stability of acene derivatives but also to retain their intrinsic optical properties, including the coveted singlet fission characteristics with a triplet yield of 158% in photovoltaics. Note that this value is considerably high and, in some cases, even better than those of common monomeric exothermic and endothermic acenes (Table S3). Furthermore, the slightly higher triplet energy of Ace-PCSi than standard TIPS-pentacene (0.80 *vs.* 0.78 eV)^114,115^ and its π-extended architecture contribute to a relatively broad and red-shifted absorption over the TIPS-pentacene analog (Fig. 2a),^113^ making Ace-PCSi an even better independent solar light absorber that holds promise to be incorporated in tandem with silicon in solar energy conversion devices. Finally, this new *cata*-π-extension approach is found to hold great promise over the previously developed *peri*-modulations in both the current toolset of synthetic protocols for stable acenes and retaining explicit photophysical characteristics for technological advancements.

### Time-resolved electron paramagnetic resonance spectroscopy

**Fig. 5:**
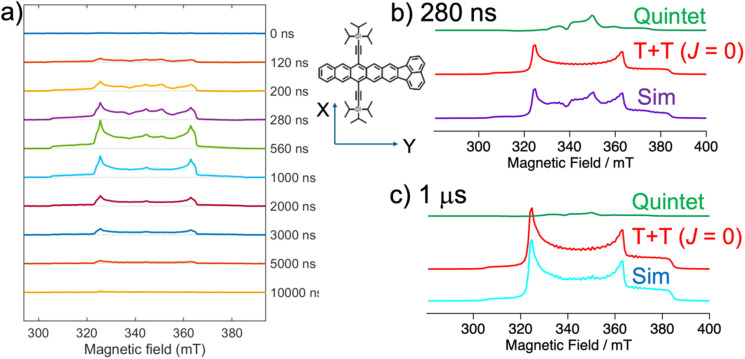
(a) Delay time dependence of the TREPR spectrum obtained by 532 nm laser irradiation of Ace-PCSi film at 80 K. The film was prepared by drop-casting a 1 mM toluene solution of 0.2 mL onto a cover glass. Microwave frequency is 9663.79 MHz. (b and c) Simulations of the TREPR spectra for the two different time regions by summations of the computed quintet and T + T hyperpolarized EPR spectra.

To further elucidate the nature of the triplet pairs and their dynamics, time-resolved electron paramagnetic resonance (TREPR) spectroscopy was employed for a neat Ace-PCSi thin film prepared with a drop casting method from a 1 mM toluene solution. Fig. 5 shows X-band TREPR spectra obtained by 532 nm laser irradiation of this film at 80 K. The microwave absorptive (*A*) hyperpolarized spectra were observed around a *g*-value of 2.003 from the microwave frequency of 9663.78 MHz, denoting that the fine structure from the triplet species is observed as a Pake pattern from *D* = 1050 MHz and *E* = 15 MHz in Table S4. In addition to the strong large fine structure exhibiting a 40 mT peak splitting, weak spike signals overlap around 330 mT and 350 mT at delay times shorter than 1000 ns. This emission (*E*)/absorption (*A*) overlapping polarization is assigned to the quintet state of the strongly coupled triplet-pair.^117^

To understand the time variation of the spectra, we performed a spin-quantum model calculation of the triplet pairs, taking into consideration the effects of the spin Hamiltonian on the exciton motion in the strongly coupled TTs (^1^TT, ^3^TT and ^5^TT) composed of TT_1_ and TT_2_ states, and in the weakly-coupled triplet pair containing the nine coupled superpositions of the basis spin functions of the diabatic ^1^(T_1_ + T_1_), ^3^(T_1_ + T_1_) and ^5^(T_1_ + T_1_) characteristics in the presence of the exchange interaction (*J*) and the external magnetic field, as reported previously.^118,119^ Table S4 summarizes the EPR and kinetic parameters to compute the hyperpolarized spectra of the quintet TT state and the separated T + T state at the delay times of 280 ns and 1 µs in (b) and (c) of Fig. 5, respectively. The absorptive feature of the spin polarization is explained by the disordered sub-nanosecond conformation dynamics of the triplet exciton within the triplet pairs. This indicates that the exciton migration of the singlet-fission-born triplets undergoes exciton migration within the pair for the singlet-quintet spin conversions to the spin sublevels (*m*_S_) of ^5^TT_*m*_S_≤0_ in the presence of the magnetic field. Summations of the quintet and T + T spectra are shown as the simulated spectra in Fig. 5b and c, which explain the experimental data in (a).

From Table S4, the singlet-precursor (^1^TT) spin interconversions occur to the quintet with the spin sublevels in ^5^TT_*m*_S_≤0_ in the presence of the magnetic field *via* the TT_2_ state, in which the T_B_ triplet in the T_A_T_B_ multiexciton takes a dihedral rotation by *β* = −76° from the TT_1_ state at the activation to weaken the exchange coupling (−11 GHz). This activation motion in the present neat thin-film sample is relevant to the triplet-exciton diffusion within the disordered solid-state environment from the strongly coupled parallel TT_1_ conformation to cause the TT_2_ state with the distorted triplet-pair conformation. At 1 µs, the absence and the presence of the quintet species and the absorptive individual triplet polarization, respectively, are explained by the preferential T + T dissociation (*k*_diss_ > *k*_back_) from the quintet species in Fig. 5c. Overall, the quintet EPR and the subsequent T + T hyperpolarization signals are the consequences of the efficient intermolecular singlet fission in a molecularly ordered crystalline environment and subsequent triplet exciton migration in the disordered region in the drop cast film. In summary, the delay time dependence of the TREPR spectrum demonstrates that singlet-precursor spin interconversions to the quintet (^5^TT) with specific spin sublevels occur from the strongly coupled triplet-pair (^1^TT), followed by triplet exciton dissociation from ^5^TT.

## Conclusions

In conclusion, we have developed a new synthetic method to linearly fuse acenaphthylene, which contains a 5-membered ring, to pentacene. In sharp contrast to other acenes stabilized through cyclopentannulation and other π-extensions, which display non-typical properties of acene and thus fail to undergo singlet fission, the acenaphtho[1,2-*b*]pentacenes Ace-PCSi and Ace-PCPh possess characteristic electronic and photophysical properties of acenes. The thin film formed from the Ace-PCSi demonstrated efficient singlet fission-mediated triplet multiexciton generation in the ultrafast time regime, with a triplet quantum yield of up to 158%. TREPR spectroscopy revealed the sequence of spin-state changes occurring during singlet fission, further confirming the formation of triplet pairs during the singlet fission process. In particular, the observation of the normally elusive quintet (^5^TT) state is notable. The impact of the acenaphthylene fusion to pentacene is further highlighted by a much longer S_1_ state and fluorescence lifetime of Ace-PCSi, and by the clear identification of a singlet contribution to the NIR absorption range with pronounced distinction between the S_1_ and T_1_ states, which have not been observed in pentacene and its derivatives.

Despite having reduced HOMO–LUMO energy gaps and elevated HOMO energy levels, linearly elongated acenaphthylene-fused pentacenes exhibit stability similar to that of their pentacene counterparts. A favorable slip-stack columnar packing pattern has been discovered in the unsymmetrical acenaphtho[1,2-*b*]pentacene Ace-PCSi, representing a notable departure from the packing behavior of other unsymmetrical acenes. This work highlights that linear fusion of acenaphthylene to acene can serve as an effective strategy to stabilize acenes while improving their photophysical properties for singlet fission. The introduction of acenaphthylene-fused acenes extends the scope of materials that can undergo singlet fission, a field previously known for its scarcity of suitable materials. The information obtained from this work will also advance the basic knowledge in understanding 4n π electron systems.

## Author contributions

H. W. and J. A. designed the project. J. A. and S. S. conducted the synthesis and characterization of the compounds. S. S. and A. W. D. carried out the initial spectral studies of the compounds. S. D. designed and carried out the experiments for transient absorption spectroscopy and data analysis. S. T. and Y. K. designed and carried out the experiments for time-resolved electron paramagnetic resonance spectroscopy. S. S. performed all the computations. V. N. N. collected the X-ray diffraction data and solved the crystal structures. S. S., T. P., and S. L. designed and conducted film preparation and measurements. All authors contributed to data analysis and manuscript writing.

## Conflicts of interest

There are no conflicts to declare.

## Supplementary Material

SC-017-D5SC08246C-s001

SC-017-D5SC08246C-s002

## Data Availability

CCDC 2450035 and 2450036 contain the supplementary crystallographic data for this paper.^120*a*,*b*^ Supplementary information (SI): experimental methods and characterization data. See DOI: https://doi.org/10.1039/d5sc08246c.
