# Supplementary material for: Linearly acenaphthylene-fused pentacene exhibiting efficient singlet fission
Source: Chem Sci. 2026 Jun 2;17(28):13997–4007. doi: 10.1039/d5sc08246c (PMC13251779; doi:10.1039/d5sc08246c)
Supplement: SC-017-D5SC08246C-s001 [file SC-017-D5SC08246C-s001.pdf]

Supplementary Information (SI) for Chemical Science.
This journal is © The Royal Society of Chemistry 2026

Supporting Information

Linearly Acenaphthylene-Fused Pentacene Exhibiting Efficient Singlet Fission

Jacob Arvidson,[‡a] Saad Shaikh,[‡a] Masahiro Tanaka,[‡c] Tejal Pawale,[b] Andrew Dawson,[a] Xiao Li,[b] Vladimir N. Nesterov,[a] Yasuhiro Kobori,[*c,d] Somnath Das,[*a] and Hong Wang[*a]

a. Department of Chemistry, University of North Texas, Denton, TX 76203, USA
b. Materials Science and Engineering Department, University of North Texas, 3940 North Elm Str, Denton, Texas 76209, United States.
c. Department of Chemistry, Graduate School of Science, Kobe University, 1-1 Rokkodaicho Nada-ku Kobe, 657-8501, Japan.
d. Laser Molecular Photoscience Laboratory, Molecular Photoscience Research Center, Kobe University, 1-1 Rokkodaicho Nada-ku Kobe, 657-8501, Japan

[‡]J. Arvidson, S. Shaikh and M.Tanaka contribute equally to this work

Table of Contents

Table of Contents 1

1 Experimental Section: 3

1.1 General description of chemicals, materials and instrumentation for synthesis 3

1.2 Spectroscopy procedures 3

1.3 Stability study of Ace-PCSi, BEP-Si, Ace-PCPh and BEPPh. 4

1.4 Transient absorption setup 4

1.5 Computational methods 5

1.6 Thin Film preparation 5

1.7 Scratch test for thickness 5

2 Synthesis Scheme 6

2.1 Synthesis Procedures 7

2.1.1 Fluoranthene-8,9-Dicarbaldehyde (Ace-Dialdehyde) 7

2.1.2 Mono-Acenaphthene Pentacene Quinone (Mono-Ace-PQ) 8

2.1.3 Mono-Acenaphthene Phenylacetylene Addition (Mono-Ace-PAA) 9

2.1.4 Mono-Acenaphthene-Pentacene 10

2.1.5 Mono-Acenaphthene (Trisisopropylsilyl) Acetylene Addition (Mono-Ace-TIPS).. 11

2.1.6 Mono-Acenaphthene-Pentacene-TIPS (Ace-PCSi) 12
3 Photophysical properties 13
3.1 Steady-state characterization 13
3.2 UV-Vis-Absorption – Experimental and Computed 14
3.3 Optical Band Gap 15
3.4 Stability Study – Ace-PCSi and BEPSi 16
3.5 Stability Study – Ace-PCPh and BEPPh 17
3.6 Fluorescence lifetime-TCSPC 18
4 Computational results 19
4.1 Kohn-Sham molecular orbitals 19
4.2 Transition Dipole Moment analysis 23
4.3 NICS (0) analysis 24
4.4 Molecular orbital energy profile 25
4.5 Excited States and Orbital Energies 26
5 Femtosecond transient absorption (fs-TA) measurement 27
6 Triplet yield calculation for Ace-PCSi 29
7 NMR 32
7.1 ^{1}H-NMR Ace-PCSi 32
7.2 ^{13}C-NMR Ace-PCSi 33
7.3 ^{1}H-NMR Ace-PCPh 34
7.4 ^{13}C-NMR Ace-PCPh 35
7.5 COSY Ace-PCSi 36
7.6 ^{1}H-NMR BEP-Si 37
7.7 ^{13}C-NMR BEP-Si 38
7.8 COSY BEP-Si 39

8 Mass- Spectrometry 40

9 X-Ray Crystallographic Data 41

10 Thin-Film-Characterization 43

10.1 Thin-Film-PXRD 43

10.2 Thin-Film thickness - AFM 44

10.3 Thin-Film thickness – Ellipsometry 45

11 List of Acenes and their respective Triplet Yields 46

12 TREPR Study 47

12 References 48

13 Author Contribution 48

1 Experimental Section:

1.1 General description of chemicals, materials and instrumentation for synthesis

Reagents were purchased from commercially available sources and used without further purification. Dry solvents were either purchased from Sigma-Aldrich or purified using commercially available solvent purification system. All the reactions were performed in standard dry glassware. Purification of compounds through column chromatography was performed using silica gel with pore size 43-63 µm or alumina. NMR spectroscopy experiments were performed using either a 500 MHz Varian NMR instrument or 400 MHz Varian NMR instrument. All NMR experiments were conducted using CDCl3, C6D6 or d8-THF as a solvent (δ 1H = 7.26 ppm; δ 13C = 77.16 ppm), (δ 1H = 7.16 ppm; δ 13C = 128.06 ppm) and (δ 1H = 3.58 ppm; δ 13C = 67.53 ppm) respectively. Mass spectrometry experiments were performed at Texas A&M University using either ESI+ or MALDI. TLC analyses were carried out on TLC plates from Silicycle (SiliaplateTM-Glass-baked-Silica). Synthesis of benzodialdehyde porphyrin and 1,2-dibromo-acenaphthylene were done using previously published procedures.

1.2 Spectroscopy procedures

All the solvents were purchased from commercial suppliers and were used by an in-house dry solvent purification system or without further purification. Steady state UV-Vis absorption was recorded using CARY 5000 spectrometer and Fluorescence were recorded using Horiba

Fluoromax-4p fluorimeter at room temperature (RT). Phosphorescence spectrum was measured (using a right-angle detection method) with a Horiba Yvon Nanolog spectrofluorometer at 77K in deoxygenated 2-methyltetrahydrofuran (MeTHF). The time-correlated single-photon counting (TCSPC) experiments were performed using Horiba-Fluoromax-4p equipped with NanoLED-605 excitation source and the decays were monitored at their respective emission maxima. AFM measurements were taken using a Bruker Dimension Icon. Film thickness was measured using a Woollam wVUV-VASE32 variable-angle spectroscopic ellipsometer.

1.3 Stability study of Ace-PCSi, BEP-Si, Ace-PCPh and BEPPh.

The half-life stability study of Ace-PCSi and BEPSi was measured in benzene solution with concentration of 0.01 mM. The solution was subjected to steady air or argon bubbling for 10 mins in the cuvette (quartz) before sealing it. The sealed cuvette was then exposed to 17W CFL lamp at room temperature keeping it 11 inches away from the lamp source. UV-Vis absorption was recorded at variable time intervals. Quartz cuvette of 1 cm path length with stopper was used. Parafilm was used to further cover stopper and sealed.

1.4 Transient absorption setup

Femtosecond transient absorption (fs-TA) measurements were performed using an ultrafast femtosecond laser by Coherent (Libra) consisting of diode-pumped mode-locked Ti:Sapphire seed laser (Vitesse) and another diode-pumped intracavity doubled Q-switched Nd:YLF laser (Evolution) to generate an amplified and compressed output laser of 800 nm (1.45 W). For the detection of optical signatures of samples, a Helios TA spectrometer coupled with a femtosecond harmonics generator, both provided by Ultrafast Systems LLC, was used. The sources for the pump and probe were derived from the fundamental output of Libra (compressed output 1.45 W, pulse width 100 fs) at a repetition rate of 1 kHz. A major portion (~95%) of this fundamental laser output (800 nm) was introduced into an optical paramagnetic amplifier system (TOPAS-Prime) for the generation of a ‘pump’ beam with a 290−2600 nm tuning range from Altos Photonics Inc., (Bozeman, MT), while the remaining output was used for the generation of a white light continuum ‘probe’ beam after traveling through delay stage (gold coated retroreflector mounted on a step motor) and finally CaF_2 crystal inside the Helios spectrometer. Kinetic traces at appropriate wavelengths were assembled from the time-resolved spectral data. All the data were chirp corrected and then analyzed using Surface Xplorer software supplied by Ultrafast Systems. Glotaran (GTA, version 1.5.1), was used for target analysis of data.[1-2] The instrument response

function of our TA setup is ~120 fs. A 630 nm pump with low pump energy (~1.46 X 10^9 photons/pulse) was maintained throughout the experiment to avoid any multiphoton absorption data and successive nonlinearity in the transient data. The probe beam was scanned both in the visible and NIR regions individually. Pump and probe beams were then tightly focused either onto a thin film or inside a 2 mm cuvette containing dilute solution of Ace-PCSi. All the measurements were performed under ambient conditions.

1.5 Computational methods

B3LYP/631G density-functional theory (DFT) was used to investigate the conformational rearrangement and energetics of Ace-PCSi, Ace-PCPh, BEPPh and BEP-Si. Geometries and frequencies were optimized using the same level of theory. Frequency calculation ensures that global minimum was calculated rather than local minimum. Kohn-Sham molecular orbitals were investigated using geometry optimized S_0 state. To calculate orbital energy at S_0, S_1, S_2 and T_1, Geometry optimized S_0 state (B3LYP/631G) was used to further optimized S_1 and T_1 state using TD-DFT function. NICS (0) study was performed by placing a dummy atom at the epicenter of the ring systems in the molecule on geometry optimized S_0, S_1 and T_1 using nmr=giao (B3LYP/631G) basis set. All the DFT and TD-DFT calculations were performed using Gaussian 16 and GaussView (6.0.16) was used as a visualization tool. [Iso-density value = 0.0004]. Transition Dipole Moment analysis was performed using Multiwfn version 3.7 and visualization was done using VMD 1.9.4a53

1.6 Thin Film preparation

A glass substrate of 1x1 cm was used to make the film. The glass substrate was washed with water and then sonicated in isopropyl alcohol to remove the impurities from the surface. After washing the substrate was treated with Piranha solution for 1 h in the fume hood. After piranha solution treatment the substrate was washed with MiliQ water several times and then heated on hot plate at 80 °C for 1 h. The glass substrate was then dried using N_2 gas. The film was prepared on the treated glass substrate using a Spin-Coater (Laurell WS-650Mz-23NPPB) at 1500 rpm for 1 min. 2% stock solution of **Ace-PCSi** in toluene was prepared and 10 µl of this solution was spin-coated on a glass substrate.

1.7 Scratch test for thickness

The thin film obtained from 2% solution was scratched using a sharp paper cutter. The film was scratched at four different sites to check the uniformity of the thickness. The film was then subjected to Atomic Force Microscopy and Ellipsometry analysis.

1.8 Time-resolved EPR measurement

Continuous wave TREPR measurements were performed using a Bruker EMXplus spectrometer. Light excitations were performed by the second harmonics (532 nm) of a Nd:YAG laser (Continuum Minilite II, fwhm = 5 ns). A light depolarizer was placed between the laser output and the optical window of the EPR cavity. Transient EPR signals were averaged by a Tektronix DPO3054 500 MHz digital phosphor oscilloscope at 201 different external magnetic field positions and were transferred to a personal computer via an USB communication to obtain the 2-dimentional TREPR data. The sample tubes were placed in an X-band cryostat system (Oxford, ESR900) with the nitrogen gas flow by a pump from the liquid nitrogen for the measurements. Temperature controller (Oxford, ITC503) was utilized to control the temperature.

2 Synthesis Scheme

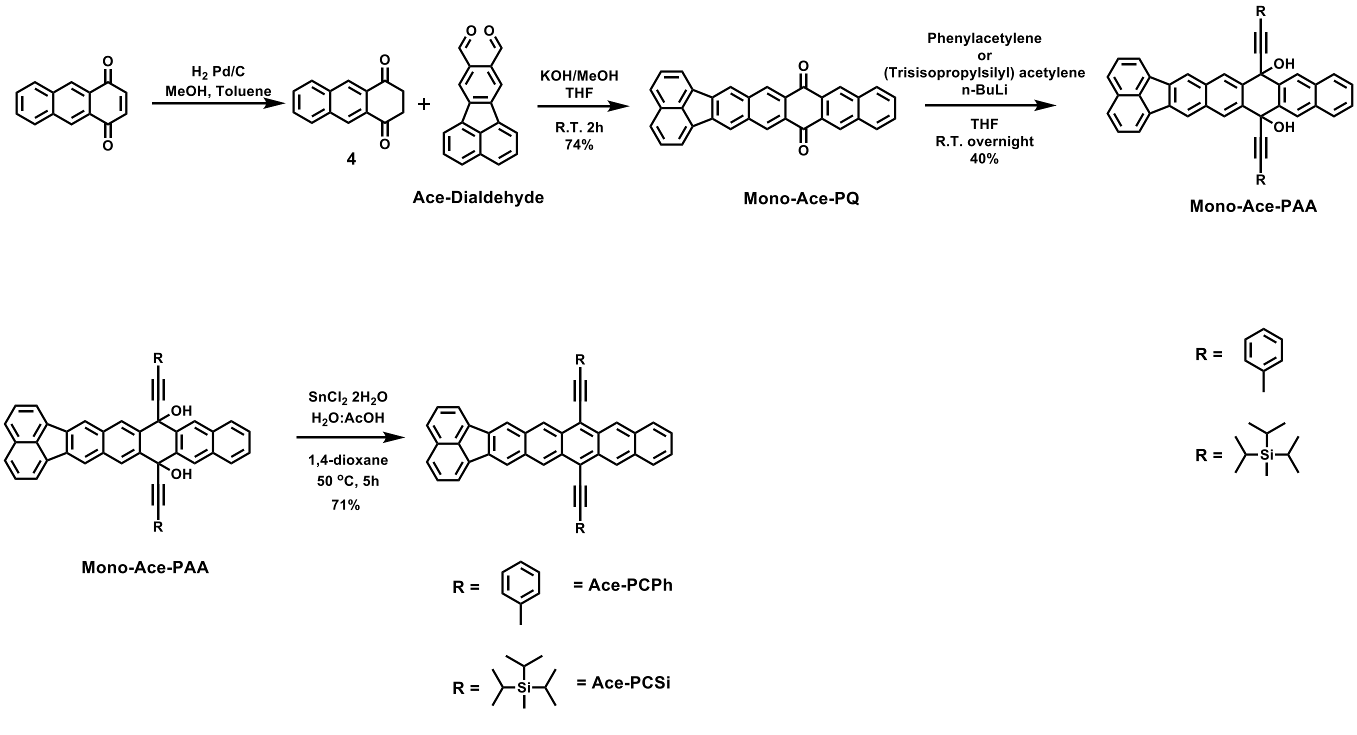

2.1 Synthesis Procedures

2.1.1 Fluoranthene-8,9-Dicarbaldehyde (Ace-Dialdehyde)

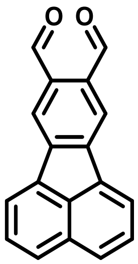

1,2-dibromoacenaphthylene (3.0 g, 9.7 mmol), acrolein diethyl acetal (8.85 mL, 58 mmol), tetrabutylammonium acetate (11.67 g, 38.8 mmol), potassium carbonate (4.01 g, 29.1 mmol), potassium chloride (1.44 g, 19.4 mmol), palladium acetate (0.27 g, 0.97 mmol), 4Å molecular sieves (5 g), and 110 mL dry DMF were added to a 500 mL round bottom flask and capped. The mixture was heated to 90 ºC for 72 h with stirring. After this period, the heating was stopped and 240 mL of 2 M HCl was cautiously added and stirring was continued for an additional 30 min. The solids were filtered and washed with a large excess of H_2O. The solids were then sonicated in water several times, filtered, and dried using reduced pressure. Dichloromethane was used to dissolve as many of the solids as possible. The solids were then filtered and placed in a mortar and pestle containing dichloromethane. The solids were ground, and the dichloromethane was decanted. This was repeated until the solvent was nearly colorless. The combined extracts were removed from the solvent under reduced pressure, leaving behind a sticky red to orange solid. The crude solids were partially dissolved in toluene, and a large excess of pentanes were added. The resulting solids were filtered and dried to yield 1.16 g of an orange solid whose 1H NMR spectrum shows only the suspected dialdehyde product. The aromatic region shows an unusual broadening ranging from 7-8.5 ppm. This polymer byproduct which strongly inhibits the sequential Aldol addition and condensation reaction can be removed by adding a small quantity of toluene, mixing well, and filtering the solids. The resulting filtrate contains mostly impurity and some product. This method yielded 0.6 g of fluoranthene-8,9-dicarbaldehyde. It should be noted that column chromatography using silica gel or alumina was not viable for purification due to stability issues.

$C_{18}H_{10}O_2$; yellow solid; yield 24%. ^1H NMR (500 MHz, $CDCl_3$) δ 10.66 (s, 2H), 8.47 (s, 2H), 8.15 (d, J = 7.0 Hz, 2H), 8.03 (d, J = 8.2 Hz, 2H), 7.82 – 7.74 (m, 2H). ^{13}C NMR (126 MHz, $CDCl_3$) δ

192.03, 143.33, 135.56, 134.54, 128.94, 128.51, 123.67, 122.72, 77.28, 77.03, 76.77; UV-Vis (CH_2Cl_2): λ_{max} = 303, 332, 380, 397 nm; HRMS (ESI+): m/z: calcd for $C_{18}H_{10}O_2$: 259.0754; found: 259.0751.

2.1.2 Mono-Acenaphthene Pentacene Quinone (Mono-Ace-PQ)

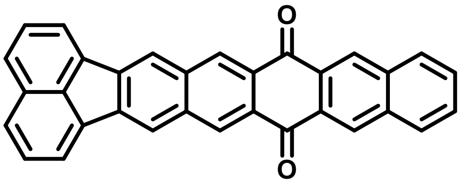

Anthracene-1,4-dione (0.1 g, 0.48 mmol) was dissolved in a mixture of MeOH:THF (50 mL: 10 mL) in a round bottom flask fitted with a two-neck adapter and stirred until solution was green in color. 10% Pd on activated carbon (0.013 g) was added to the round bottom flask and simultaneously fitted with a H_2 balloon on one neck and connected to a vacuum line with the other neck. H_2 was evacuated and backfilled into the reaction flask five times using vacuum. The reaction was stirred at room temperature for 2 h until the solution remains red in color. Into a Schlenk flask was added Ace-Dialdehyde and THF. The solution was degassed by bubbling argon for 15 min. In a 20 mL vial 0.15M KOH/MeOH (9.6 mL, 0.0014 mmol) was degassed by bubbling argon for 15 min. The reduced anthracene **4** was transferred to the acenaphthene dialdehyde solution using a syringe fitted with a 0.22 µm PTFE filter under strictly argon atmosphere. The KOH/MeOH solution was then added dropwise over 5 min under argon atmosphere with vigorous stirring. After 30 min, the precipitated solid was filtered and washed well with THF and MeOH to yield 0.123 g of the insoluble acenaphthene-fused pentacene quinone.

$C_{32}H_{16}O_2$; orange solid; yield 74%. Compound could not be characterized due to insolubility in common organic solvents.

2.1.3 Mono-Acenaphthene Phenylacetylene Addition (Mono-Ace-PAA)

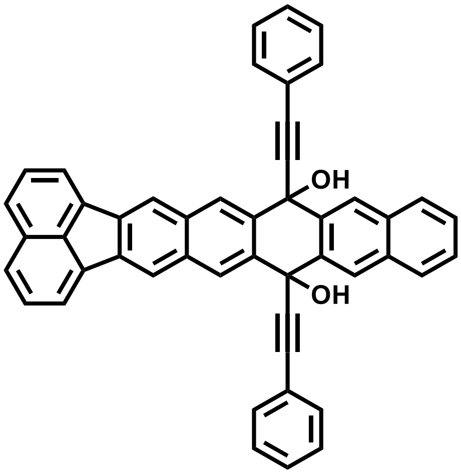

10 mL THF was added to a Schlenk flask under argon and cooled to -78 °C in a dry ice-acetone bath. Lithium phenyl acetylide was prepared in situ by sequentially adding phenylacetylene (1.37 mL, 12.51 mmol) and 2.5 M n-butyl lithium solution (4.5 mL, 11.38 mmol) to the cooled THF flask. In a separate Schlenk flask was added Mono-Ace-PQ (0.123 g, 0.284 mmol). The flask was placed under the argon and 5 mL THF was added. The lithium phenyl acetylide solution was slowly added to the quinone flask and stirred well for 12 h. Once the reaction was complete the solvent was removed under reduced pressure. The solids were dissolved in dichloromethane and washed well with water three times. The organic portion was collected with a separatory funnel, and the solvent was removed under reduced pressure. The solids were dissolved in a minimal amount of toluene (10 mL) and hexanes were added (20 mL) to crash out the crude product. The resulting solids were run through a short alumina column using 2:1 (dichloromethane: hexanes) as the eluent to isolate the most non-polar highly fluorescent UV active band. The solvent was removed under reduced pressure to obtain 0.0723 g pure of the desired product.

$C_{48}H_{28}O_2$; pale yellow solid; yield 40%, ^{1}H NMR (500 MHz, $CDCl_3$) δ 8.79 (s, 2H), 8.73 (s, 2H), 8.43 (s, 2H), 8.06 (d, 2H), 7.99 (dd, 2H), 7.87 (d, 2H), 7.69 (dd, 2H), 7.56 (m, 6H), 7.35 (m, 6H), 3.55 (s, 2H).

2.1.4 Mono-Acenaphthene-Pentacene

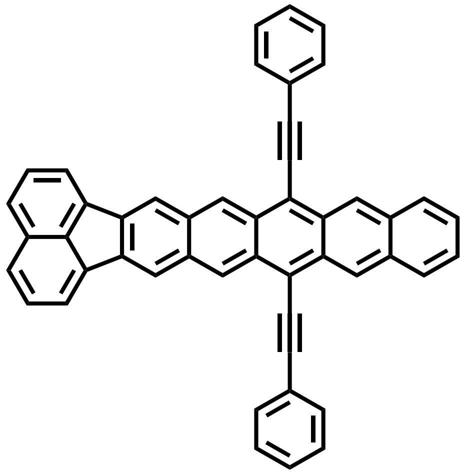

A suspension of tin chloride dihydrate (0.12 g, 2.2 mmol) in 0.5 mL of glacial acetic acid and 0.5 mL H_2O was prepared. Mono-Ace-PAA (0.040 g, 0.06 mmol) was dissolved in 12 mL of 1,4-dioxane and pipetted into the tin chloride suspension. The mixture was heated at 50 ^{o}C for 3 h protected well from light. After this period 10 mL of H_2O was added. The resulting solids were filtered, washed well with H_2O, and dried in darkness under reduced pressure. The solids were then dissolved in THF and the insoluble were removed by filtration. The solvent was mostly removed under reduced pressure and hexanes were added to crash out the product. The resulting solids were filtered to obtain pure mono-acenaphthene pentacene as a metallic appearing green-black solid.

$C_{48}H_{26}$; black-green solid; yield 71%, ^{1}H NMR (500 MHz, d8-THF) δ 9.33 (d, 4H), 8.62 (s, 2H), 8.11 (m, 4H), 8.0 (d, 4H), 7.88 (d, 2H), 7.71 (dd, 2H), 7.58 (dd, 4H), 7.53 (d, 2H), 7.45 (dd, 2H); ^{13}C NMR (126 MHz, d8-THF) δ 139.56, 138.27, 137.75, 133.61, 133.59, 132.90, 132.22, 131.46, 131.17, 129.93, 129.75, 129.63, 129.42, 127.84, 127.16, 127.03, 126.99, 124.79, 121.56, 119.84, 119.01, 105.74, 88.57; UV-Vis (Toluene): λ_{max} = 380, 402, 462, 496, 570, 620, 678 nm; HRMS (MALDI): m/z: calcd for $C_{48}H_{26}$: 602.204; found: 602.104.

2.1.5 Mono-Acenaphthene (Trisisopropylsilyl) Acetylene Addition (Mono-Ace-TIPS)

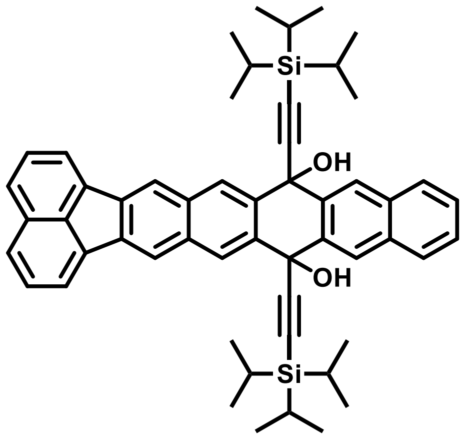

10 mL THF was added to a Schlenk flask under argon and cooled to -78 °C in a dry ice-acetone bath. Lithium (Trisisopropylsilyl) acetylide was prepared in situ by sequentially adding (Trisisopropylsilyl) acetylene (0.39 mL, 1.734 mmol) and 2.5 M n-butyl lithium solution (0.55 mL, 1.382 mmol) to the cooled THF flask. In a separate Schlenk flask was added Mono-Ace-PQ (0.05 g, 0.1156 mmol). The flask was placed under the argon and 5 mL THF was added. Lithium (Trisisopropylsilyl) acetylide solution was slowly added to the quinone flask and stirred well for 12 h. Once the reaction was complete 2 ml of 10% Hydrochloric Acid was added to reaction mixture the THF solvent was removed under reduced pressure. The solids were dissolved in chloroform and washed well with water three times. The organic layer turns orange-brown in color. The organic layer was collected with a separatory funnel and the solvent was removed under reduced pressure. The solid was used for the next step without further purification.
$C_{54}H_{60}O_2Si_2$; orange-brown solid.

2.1.6 Mono-Acenaphthene-Pentacene-TIPS (Ace-PCSi)

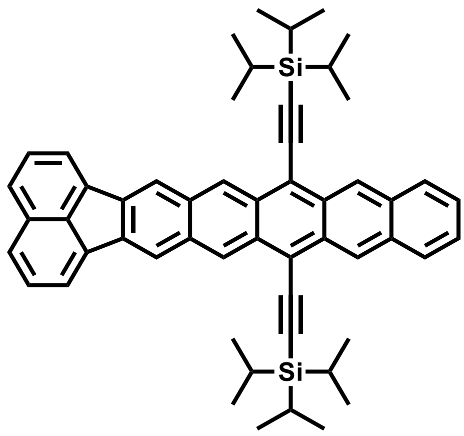

A suspension of tin chloride dihydrate (0.026 g, 0.11 mmol) in 1.5 mL of glacial acetic acid and 1.5 mL H_2O was prepared. Mono-Ace-TIPS (0.092 g, 0.11 mmol) was dissolved in 6 mL of Acetone and pipetted into the tin chloride suspension. The mixture was stirred 24 h at room temperature protected well from light. After the reaction is done, the solvent was removed under reduced pressure in rotavap. The solid was dissolved in dichloromethane and was worked up three times using sat. $NaHCO_3$ and later with water three times. The workup was performed under dim light. The organic layer was collected and dry under reduced pressure in rotavap and then further subjected to alumina column using (dichloromethane: hexane) (1:3). The concentration of Dichloromethane was further increased to get the green band out of column. The solvent was dried and solid was added in Acetonitrile. The solid was filtered and the resulting solid was greenish-black in color.

$C_{54}H_{58}Si_2$; black-green solid; yield 40%, 1H NMR (500 MHz, alumina filtered $CDCl_3$) δ 9.33 (d, 4H), 8.39 (s, 2H), 8.094 (d, 2H), 7.98 (dd, 2H), 7.86 (d, 2H) 7.71 (t, 2H), 7.42 (dd, 2H), 1.47 (m, 42); ^{13}C NMR (126 MHz, $CDCl_3$) δ 138.41, 137.26, 136.65, 132.39, 132.33, 131.02, 130.98, 130.78, 128.83, 128.45, 127.24, 126.47, 126.13, 120.51, 118.84, 118.44, 107.18, 104.77,19.23, 11.87; UV-Vis (Toluene): λ_{max} = 330, 385, 401, 436,458, 493, 561, 606, 664 nm; HRMS (ESI): m/z: calcd for $C_{48}H_{26}$: 762.4072; found: 762.4053.

3 Photophysical properties

3.1 Steady-state characterization

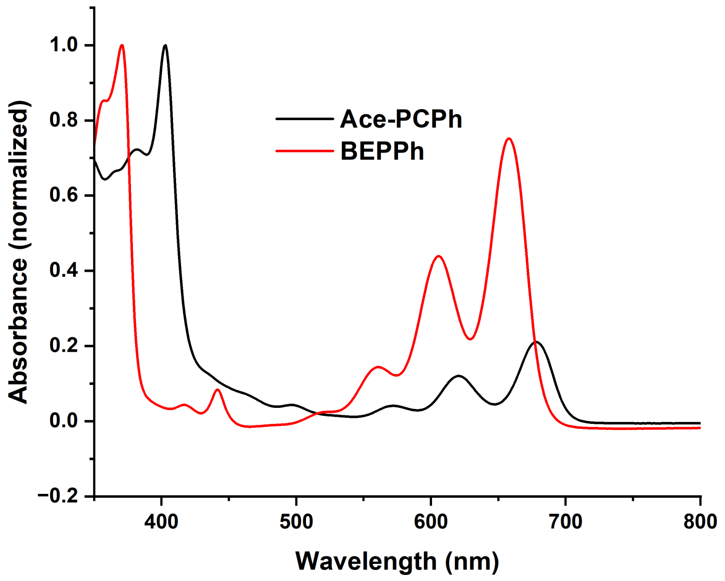

Figure S1. Absorption spectra (top) of **Ace-PCPh** (**black**) and **BEP-Ph** (**red**) in toluene**.**

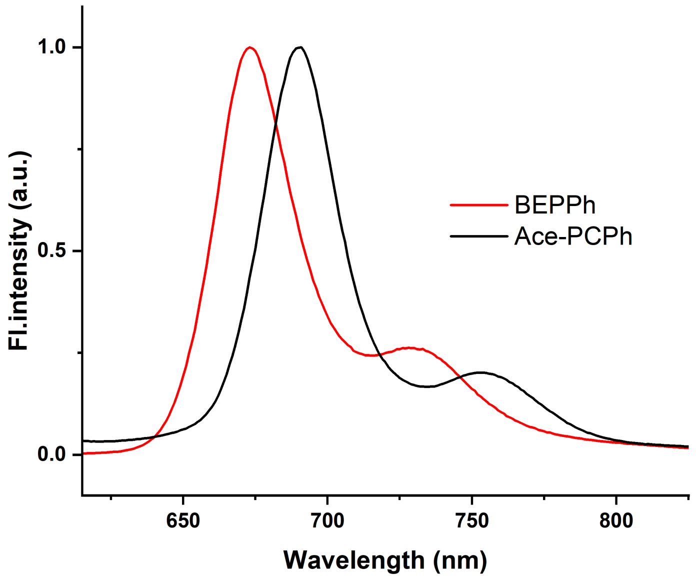

Figure S2. Emission spectra (top) of **Ace-PCPh** (**black**) and **BEP-Ph** (**red**) in toluene

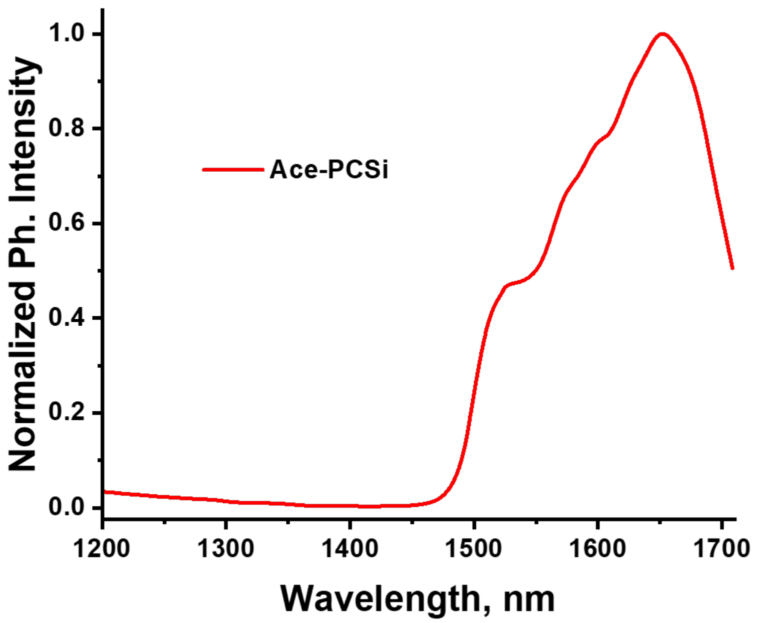

Figure S3. Phosphorescence spectrum (top) of **Ace-PCSi** in deoxygenated 2-Methyltetrahyfrofuran at 77K.

3.2 UV-Vis-Absorption – Experimental and Computed

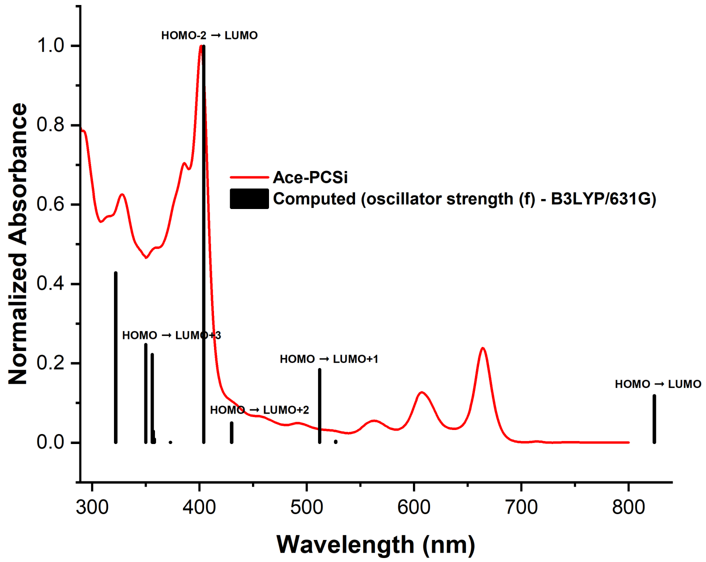

Figure S4. Absorption spectra (**red**) and TDDFT (B3LYP/631G) computed oscillator strength of **Ace-PCSi** (**black**).

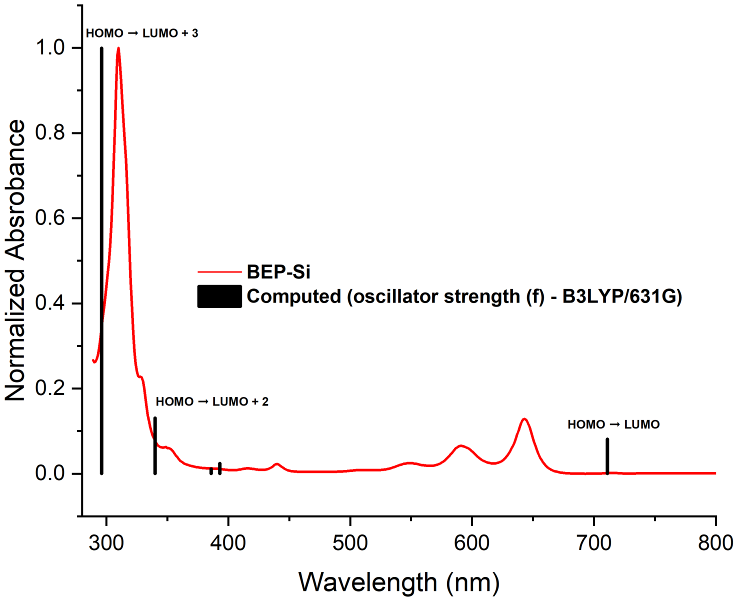

Figure S5. Absorption spectra (**red**) and TDDFT (B3LYP/631G) computed oscillator strength of **BEP-Si** (**black**).

3.3 Optical Band Gap

**Compounds**	UV-Vis **(onset)**	**(Optical gap)** $E_g^{opt} \approx E_{LUMO}-E_{HOMO}$
**BEP-Si**	669 nm	1.85 eV
**Ace-PCSi**	688 nm	1.80 eV

Table S1. HOMO-LUMO estimated through optical gap calculated experimentally derived from **onset** of lowest energy electronic transition in absorption profile.

3.4 Stability Study – Ace-PCSi and BEPSi

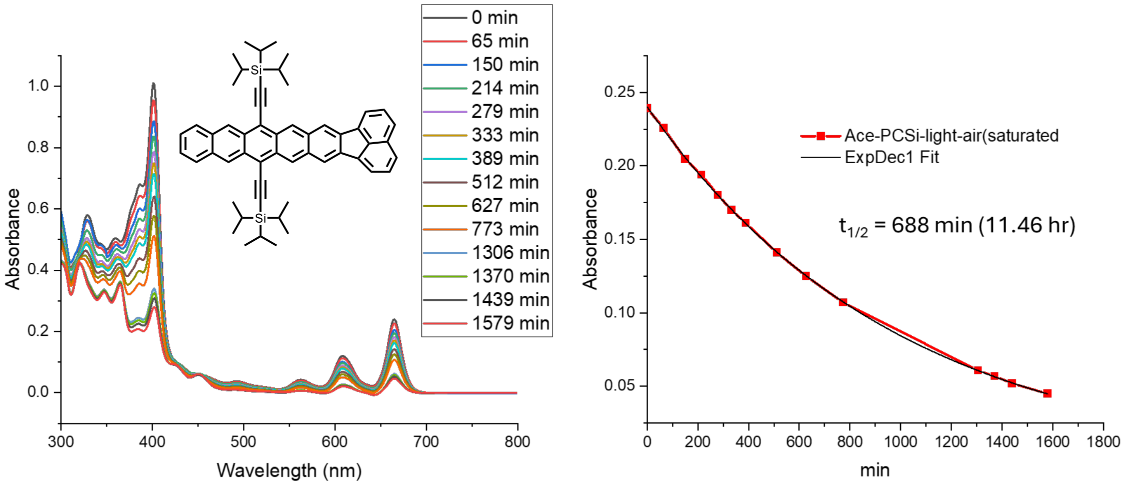

Figure S6. Stability study (top) of **Ace-PCSi** in 0.01 mM benzene (air saturated) solution under 17W CFL lamp kept 11 inches away from sample (half-life evaluated using single exponential fit).

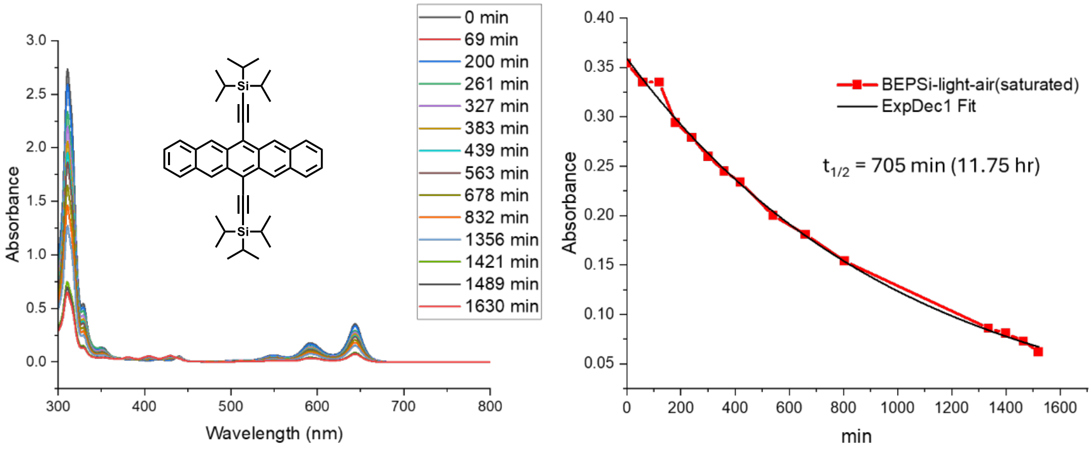

Figure S7. Stability study of **BEPSi** in 0.01 mM benzene (air saturated) solution under 17W CFL lamp kept 11 inches away from sample (half-life evaluated using single exponential fit).

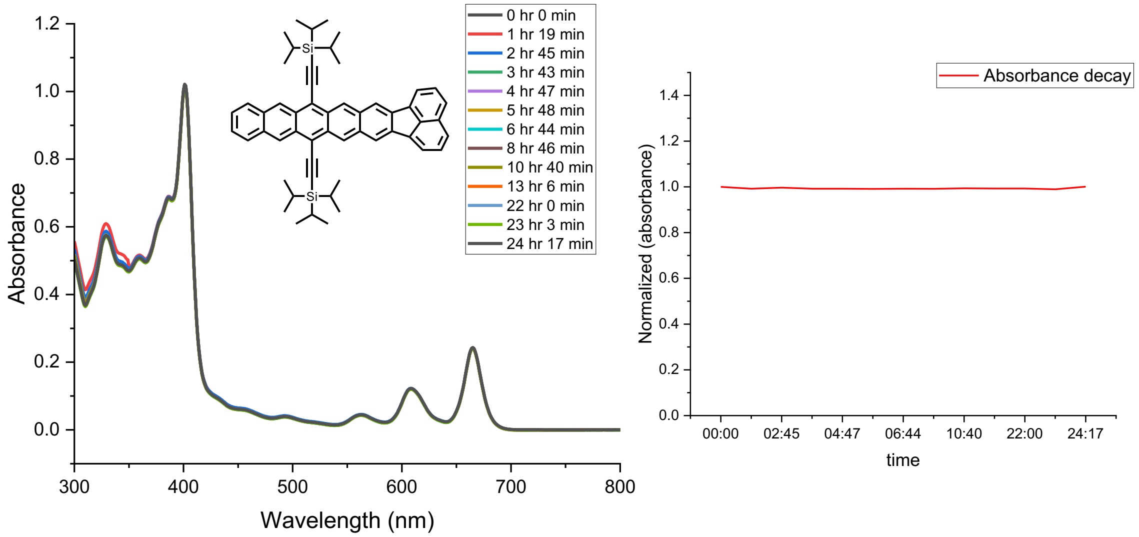

Figure S8. Stability study (top) of **Ace-PCSi** in 0.01 mM benzene (air saturated) solution kept in the **dark**.

3.5 Stability Study – Ace-PCPh and BEPPh

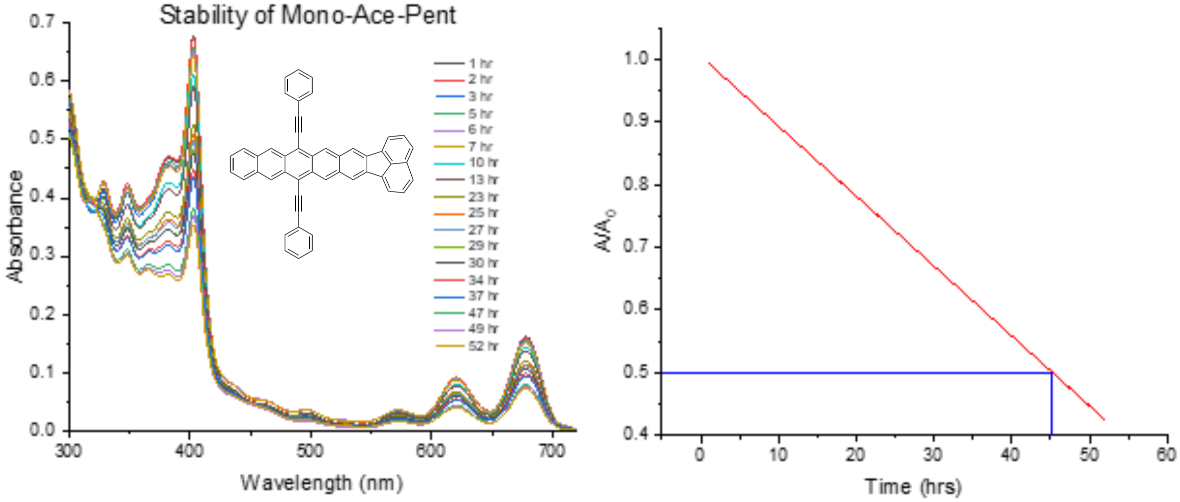

Figure S9. Stability study of **Ace-PCPh** in 0.01 mM benzene solution (no air saturation) under 17W CFL lamp kept 11 inches away from sample.

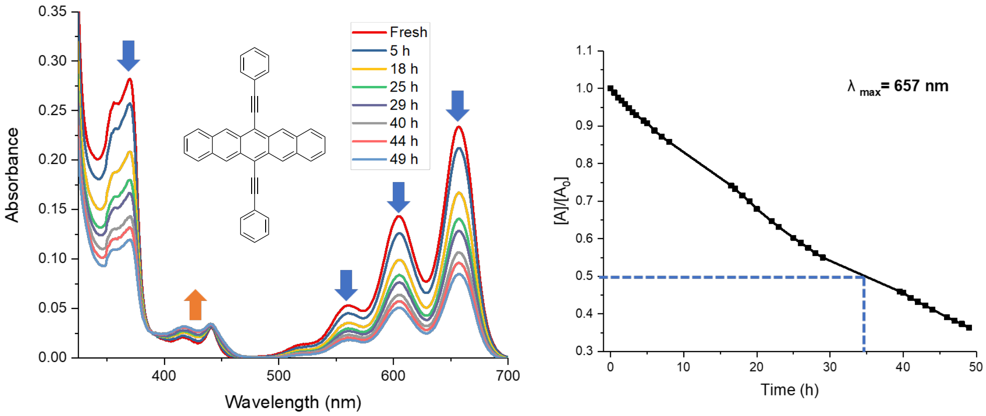

Figure S10. Stability study of **BEPPh** in 0.01 mM benzene solution (no air saturation) under 17W CFL lamp kept 11 inches away from sample.

3.6 Fluorescence lifetime-TCSPC

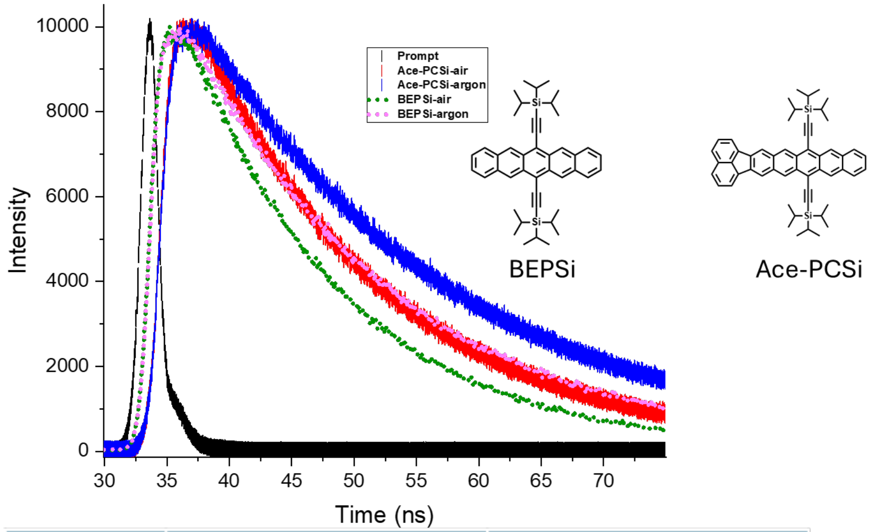

Lifetime (ns)	Air (ns)		Argon (ns)	
	Trial 1	Trial 2	Trial 1	Trial 2
Ace-PCSi	15.0	15.3	20.2	20.4
BEPSi	12.5	12.6	16.4	17.3

Figure S11. Measured TCSPC decay (lifetime) curve recorded for **Ace-PCSi** and **BEPSi** in toluene with air and argon purging (10 min) and lifetime calculated using single exponential decay fit (Excited with NanoLED 605 nm and monitored at emission wavelength of 669 nm and 649 nm)

4 Computational results

4.1 Kohn-Sham molecular orbitals

Ace-PCSi

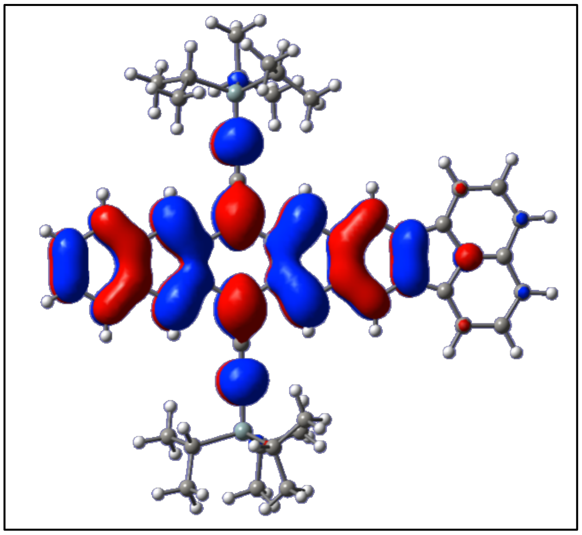

LUMO (-2.65 eV)

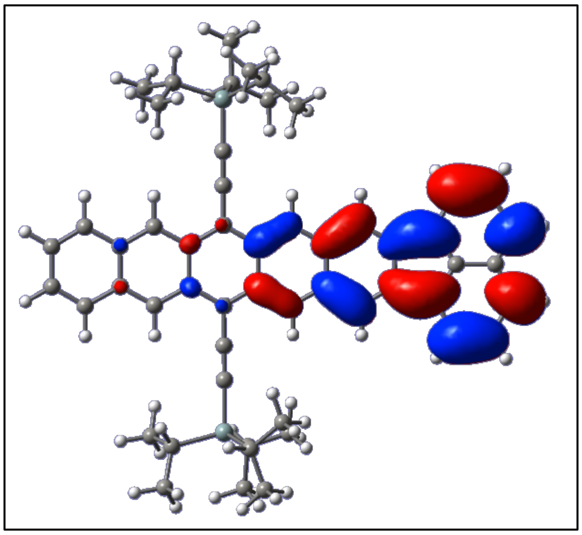

LUMO+1 (-1.66 eV)

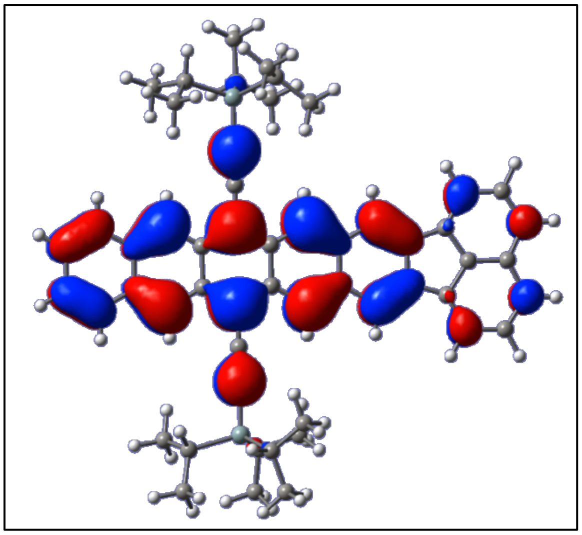

HOMO (-4.55 eV)

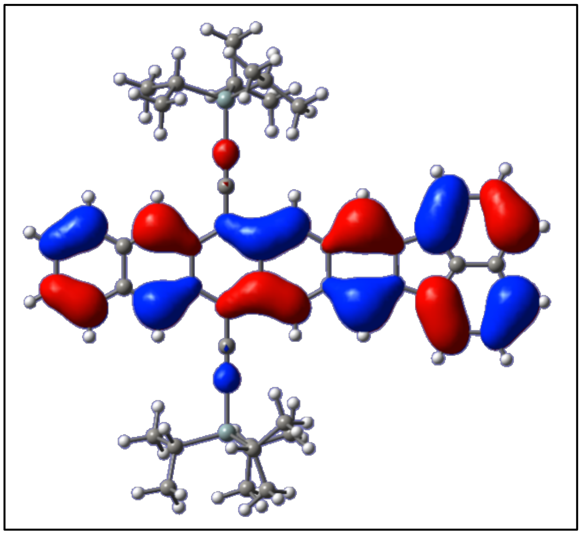

HOMO-1 (-5.58 eV)

Figure S12. Kohn-Sham HOMO-1, HOMO, LUMO and LUMO+1 molecular orbital (top) of **Ace-PCSi**, DFT optimized geometries at B3LYP/631G level in S_0.

Ace-PCPh

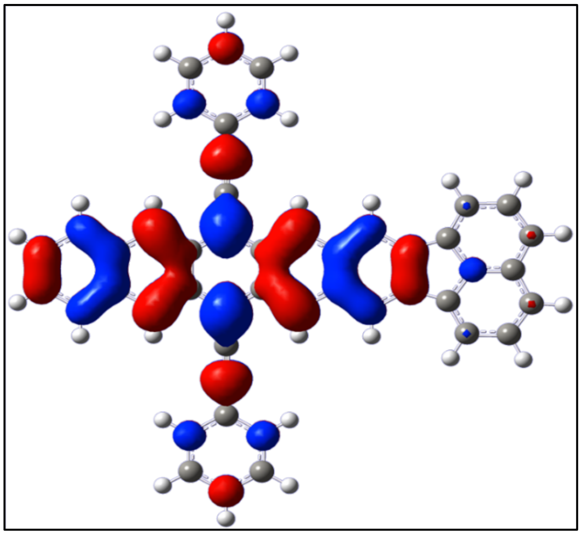

LUMO (-2.72 eV)

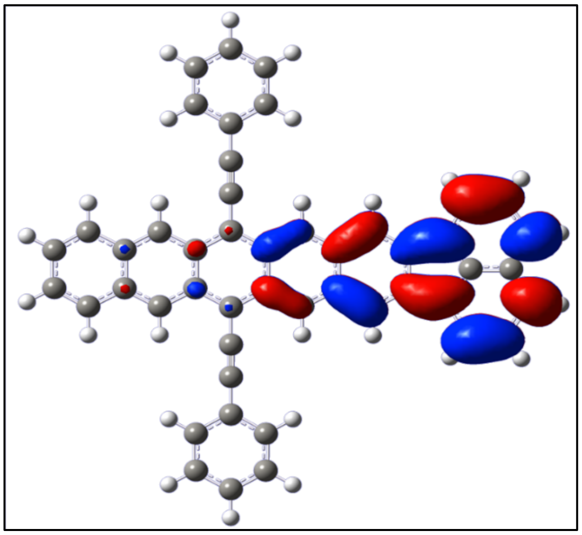

LUMO+1 (-1.69 eV)

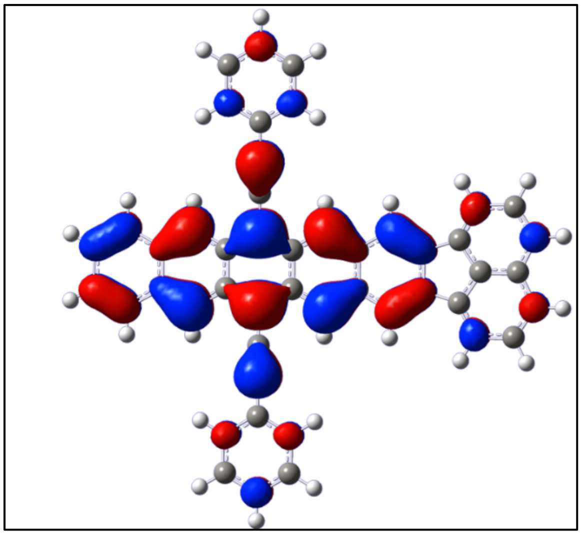

HOMO (-4.51 eV)

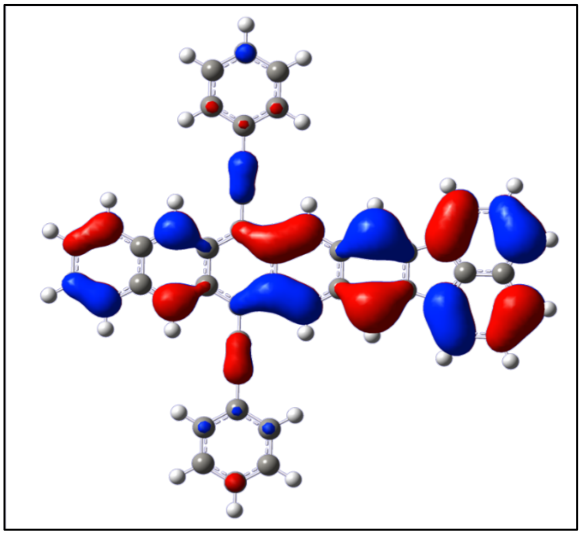

HOMO-1 (-5.58 eV)

Figure S13. Kohn-Sham HOMO-1, HOMO, LUMO and LUMO+1 molecular orbital (top) of **Ace-PCPh**. DFT optimized geometries at B3LYP/631G level in S_0.

BEP-Si

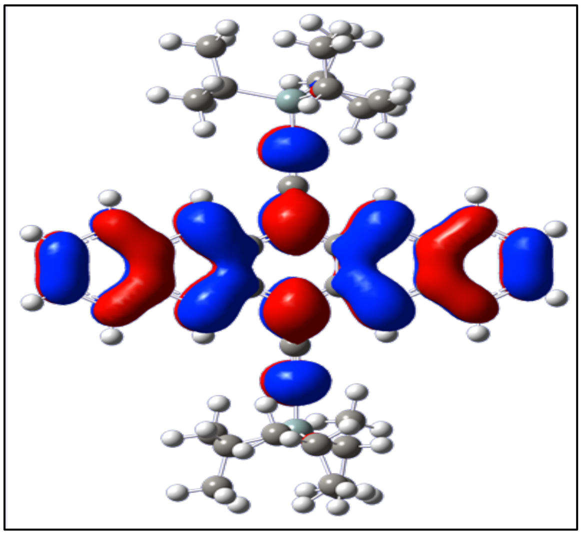

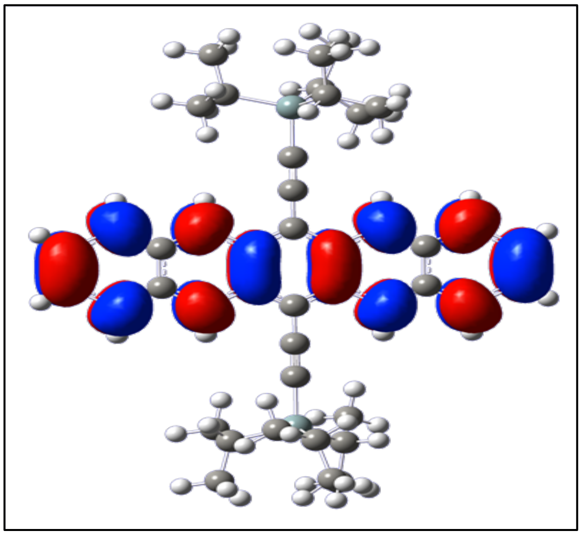

LUMO (-2.64 eV) **LUMO+1 (-0.93 eV)**

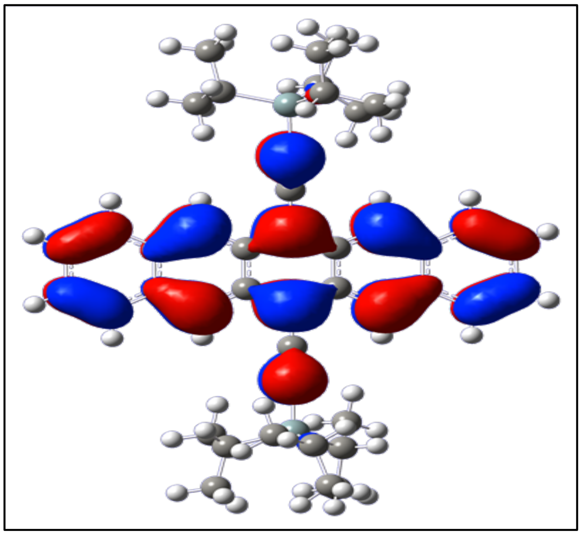

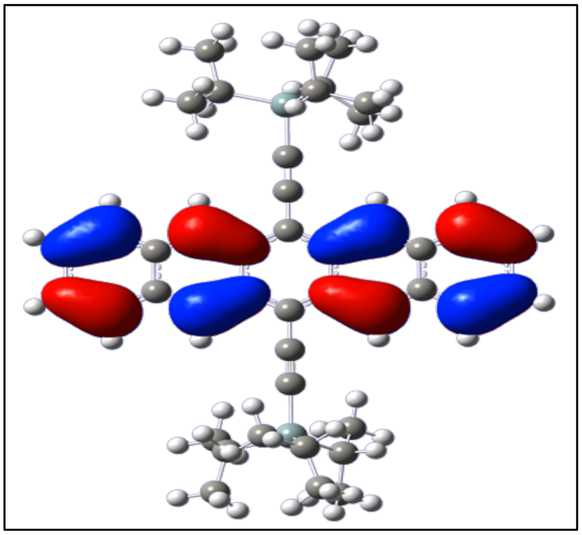

HOMO (-4.57 eV) **HOMO-1 (-5.90 eV)**

Figure S14. Kohn-Sham HOMO-1, HOMO, LUMO and LUMO+1 molecular orbital (top) of **BEP-Si**, DFT optimized geometries at B3LYP/631G level in S_0.

BEPPh

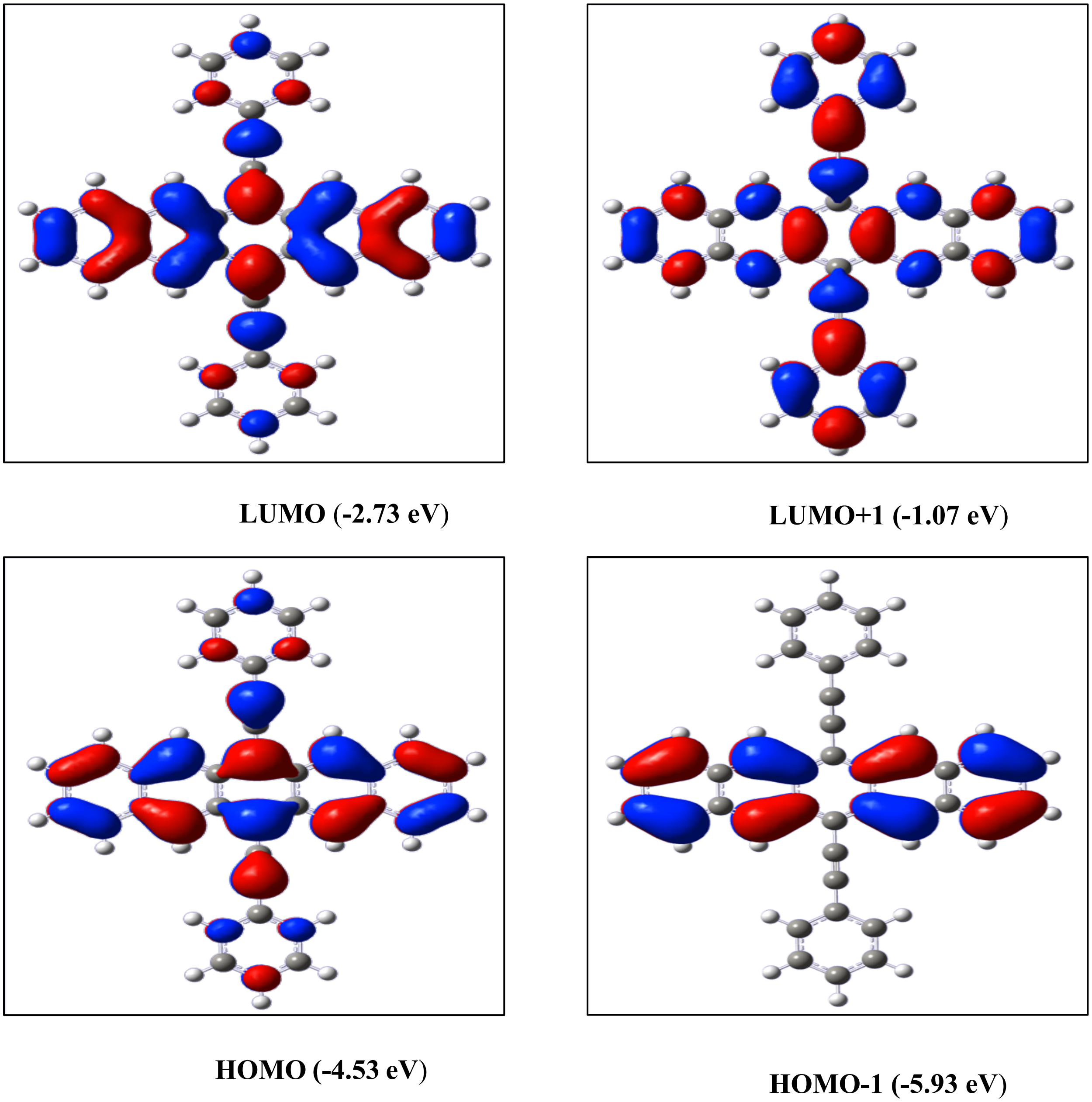

Figure S15. Kohn-Sham HOMO-1, HOMO, LUMO and LUMO+1 molecular orbital (top) of **BEPPh,** DFT optimized geometries at B3LYP/631G level in S_0.

4.2 Transition Dipole Moment analysis

Transition dipole moment- Basis Set (B3LYP/631G)-Ace-PCSi

a)

	Ace-PCSi-B3LYP/631G			
State	Excited State (nm)	Energy (eV)	Osc. Strength (f)	|µ| (Debye)
S1	824.86	1.503	0.1362	9.4
S2	527.67	2.35	0.0219	0.97
S3	512.51	2.419	0.2018	8.66
S4	430.42	2.881	0.0674	2.43
S5	421.49	2.942	0.0033	0.12
S6	404.72	3.063	1.0122	34.3
S7	373.06	3.323	0.0191	0.6
S8	358.29	3.461	0.027	0.81
S9	357.71	3.466	0.0461	1.38
S10	356.61	3.477	0.2393	7.14
S11	351.06	3.532	0.0173	0.51
S12	350.32	3.539	0.2644	7.75
S13	343.01	3.615	0.0003	0.008
S14	330.69	3.749	0.0004	0.012
S15	322.26	3.847	0.4444	11.98

b)

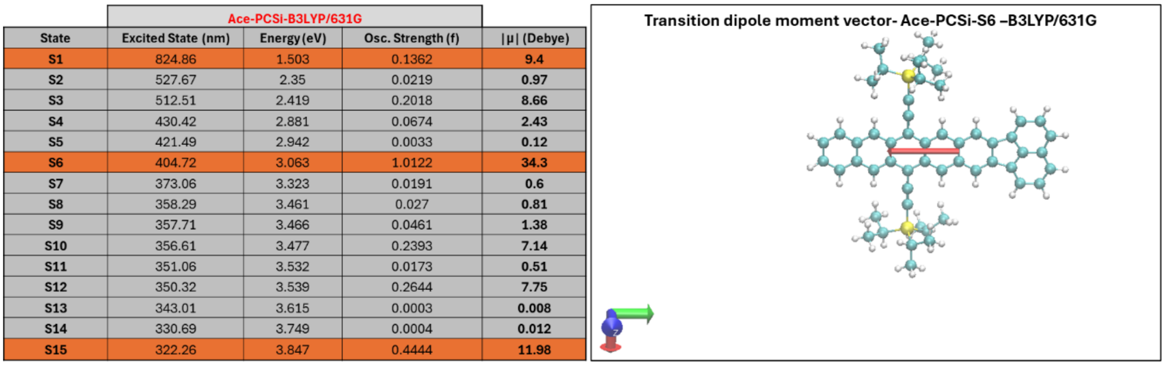

Figure S16 Excited states and their photophysical properties **(a)** and Transition dipole moment vector analysis of excited state **6** of **Ace-PCSi (b),** Analyze and visualize using Multiwfn version 3.7[1] and VMD 1.9.4a53.

Transition dipole moment- Basis Set (B3LYP/631G)-BEP-Si

a)

	BEP-Si-B3LYP/631G			
State	Excited State (nm)	Energy (eV)	Osc. Strength (f)	|µ| (Debye)
S1	710.99	1.744	0.1909	11.36
S2	453.39	2.735	0	0
S3	392.83	3.156	0.0676	2.22
S4	387.32	3.201	0	0
S5	385.85	3.213	0.0381	1.23
S6	345.54	3.588	0.0013	0.04
S7	340.77	3.638	0.0023	0.07
S8	339.9	3.648	0.2996	8.52
S9	332.32	3.731	0.0007	0.02
S10	298.4	4.155	0.0023	0.06
S11	295.7	4.193	2.1957	54.32
S12	292.78	4.235	0.0125	0.31
S13	282.45	4.39	0.0003	0.01
S14	279.83	4.431	0.0013	0.03
S15	275.39	4.502	0.0006	0.01

b)

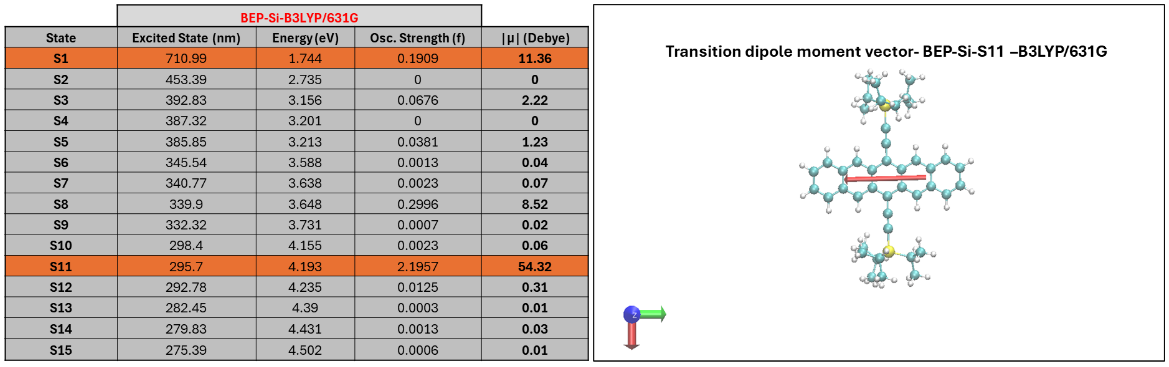

Figure S17 Excited states and their photophysical properties **(a)** and Transition dipole moment vector analysis of excited state **11** of **BEP-Si (b)**, Analyzed and visualized using Multiwfn version 3.7[1] and VMD 1.9.4a53

TDM analysis

The transition dipole moment (TDM) analysis of Ace-PCSi and BEP-Si reveals distinct electronic behaviors. In both compounds, the TDM vectors lie predominantly in the xy plane, consistent with π–π* transitions as shown if Figure S16 and Figure S17b. In BEP-Si excited state **S_{11}** exhibits a larger transition dipole (54.32 D) while in **Ace-PCSi** it is **S_6** (34.3 D). Notably, these states

correspond to the brightest transitions with the highest oscillator strengths for both molecules. The most intense transition of **Ace-PCSi** occurs at 404 nm, whereas **BEP-Si** absorbs at 295 nm, indicating a significant red shift for **Ace-PCSi**, which aligns well with the experimental data. This red shift arises primarily from the cata-annulation of the acenaphthylene unit onto the pentacene and thereby extending the conjugation.

4.3 NICS (0) analysis

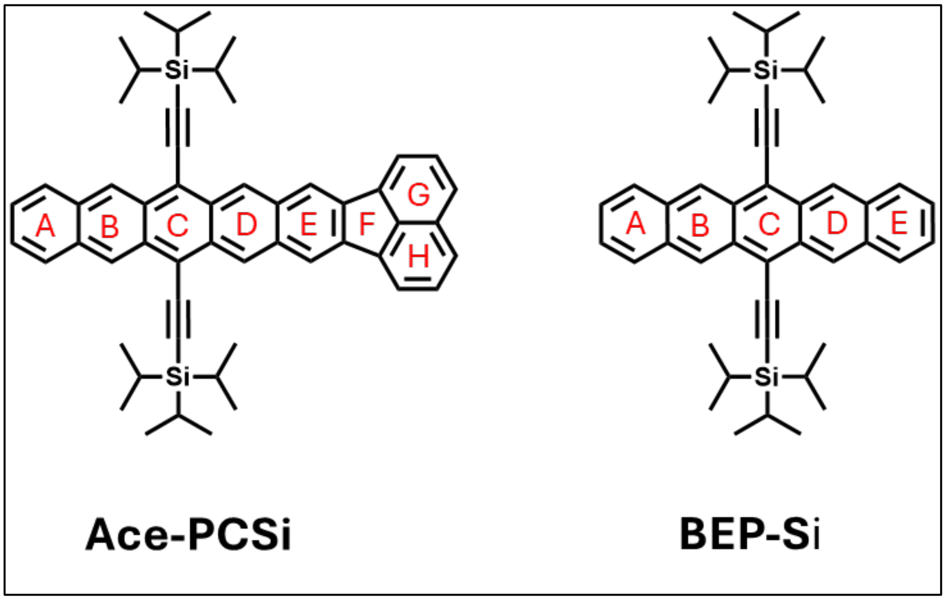

NICS 0 (nmr = giao, B3LYP/631G)								
S_0	A	B	C	D	E	F	G	H
Ace-PCSi	-5.46	-9.52	-9.37	-8.27	-3.61	4.60	-6.24	-6.26
BEP-Si	-5.36	-9.62	-9.90	-9.59	-5.46			
T_1	A	B	C	D	E	F	G	H
Ace-PCSi	-5.00	-1.47	4.97	-1.13	-3.23	4.73	-6.14	-6.20
BEP-Si	-5.07	-1.61	4.90	-1.60	-5.05			
S_1	A	B	C	D	E	F	G	H
Ace-PCSi	-6.51	-9.64	-8.78	-8.36	-4.34	4.97	-6.10	-6.16
BEP-Si	-6.46	-9.36	-9.15	-9.71	-6.47			

Table S2. NICS (0) analysis performed on **Ace-PCSi** and **BEP-Si** at DFT/TDDFT optimized geometries (S_0,S_1, and T_1) using nmr = giao B3LYP/631G level.

4.4 Molecular orbital energy profile

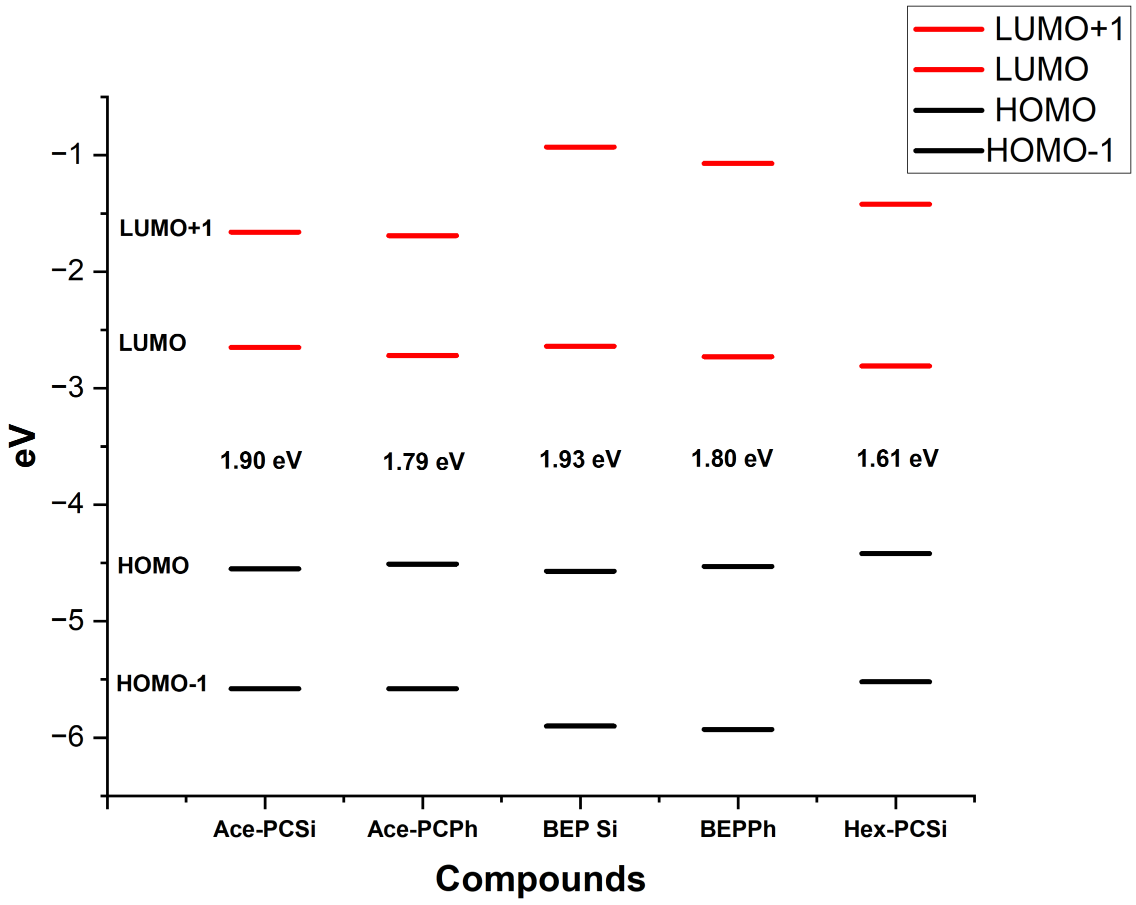

Figure S18. Energy levels (top) of **Ace-PCSi**, **Ace-PCPh**, **BEP-Si**, **BEP-Ph** and **Hex-PCSi**. DFT optimized geometries at B3LYP/631G level in S_0.

4.5 Excited States and Orbital Energies

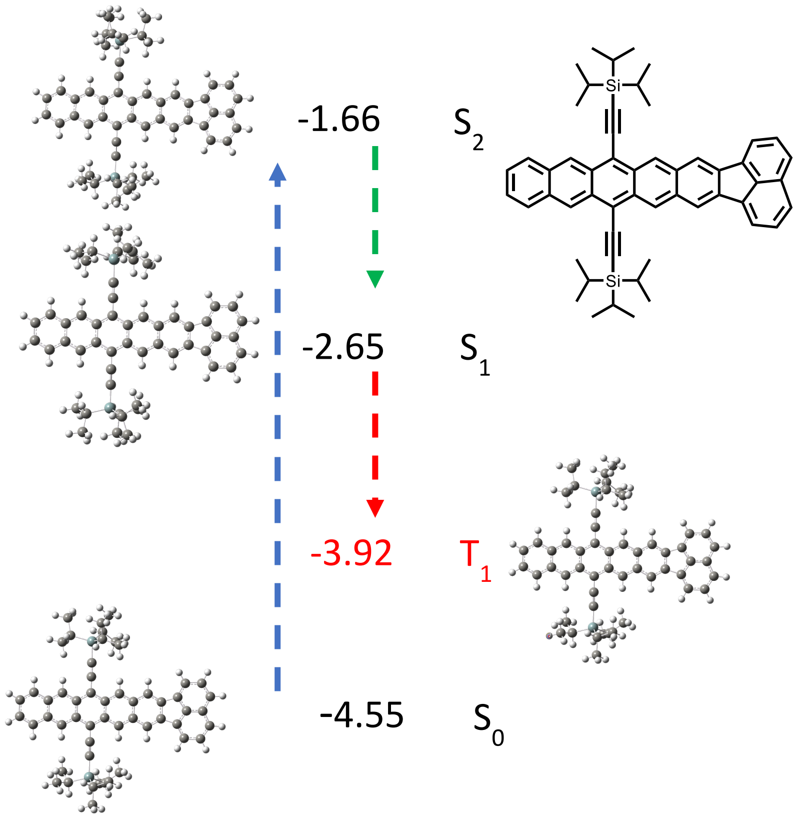

Figure S19. Energy levels (top) of **Ace-PCSi**. DFT and TD-DFT optimized geometries at B3LYP/631G level for S_0, S_1, S_2 and T_1 for calculations at the same level.

5 Femtosecond transient absorption (fs-TA) measurement

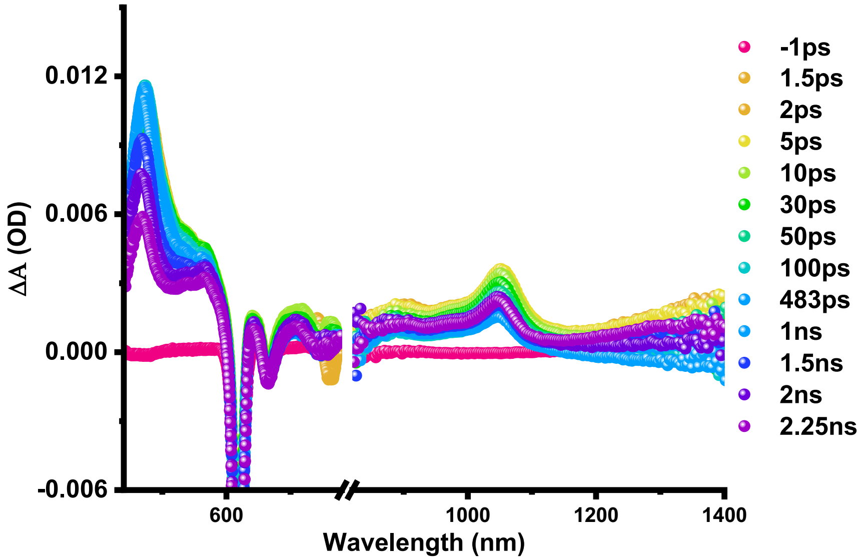

Figure S20. TA spectra (top) of **Ace-PCSi** at different delay times in toluene (concentration ~ 0.01 mM) obtained upon excitation at 630 nm and showing dominant singlet bands at ~440-500 nm in the visible and ~800-1100 nm in the NIR.

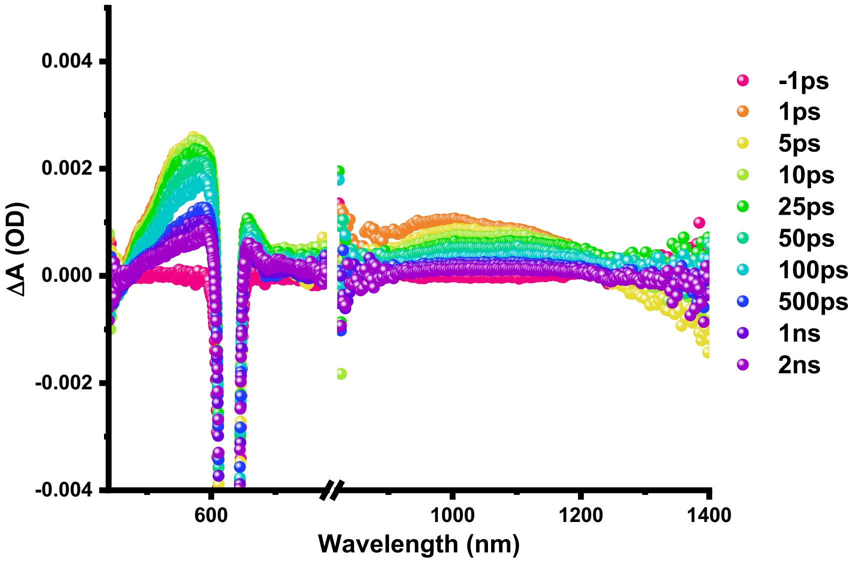

Figure S21. TA spectral evolutions (top) of **Ace-PCSi** thin film at longer (>1 ps) time window (λ_{ex}= 630 nm) in the visible and NIR regions.

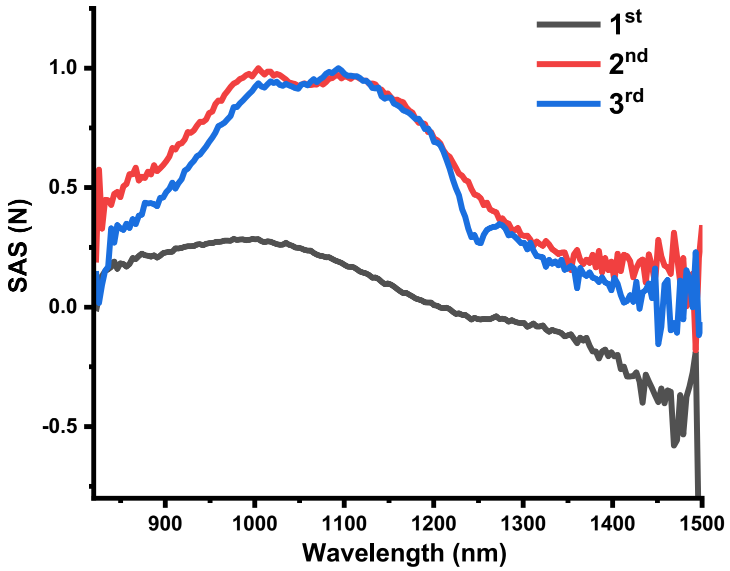

Figure S22. SAS (top) of **Ace-PCSi** obtained after a sequential three-component GTA fitting in the NIR region. A similar second and third component suggests that no more intermediate species could be retrieved after the second one.

6 Triplet yield calculation for Ace-PCSi

❖ Thickness of the Ace-PCSi thin film= ~10 nm. Note that two different techniques, AFM and Ellipsometry are used to obtain thickness of the film and the outcome of both studies are found to be comparable (Figure S22 and S23).

❖ The unit cell density of crystalline Ace-PCSi obtained from single crystal XRD measurement is 1.15 g/cm^3, that yields a concentration of the thin film ~ 1.51 M. Interestingly, the density is found to be quite similar to that reported in TIPS-Pentacene (1.104 g/cm^3),[4] suggesting linear π-extension do not impact much to the unit cell crystal structure of pristine pentacene system.

❖ Optical density of lowest energy absorption at 684 nm is ~0.028

❖ Ground state molar absorptivity at 684 nm = 18543.0 M^{-1} cm^{-1}.

Since ultrafast (<1 ps) singlet to triplet pair formation takes place in Ace-PCSi, it is thus expected that the species present in ps time window mostly represent the triplets. We have therefore considered 40 ps delay time TA data (shown below) for the calculation of triplet concentration and triplet absorptivity upon photoexcitation.

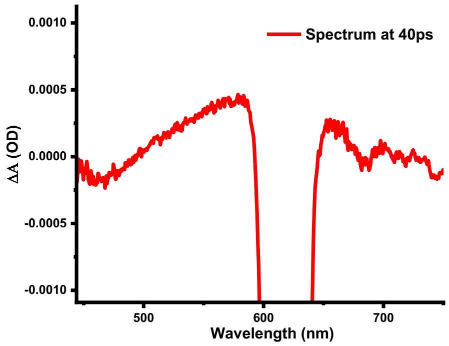

❖ Amplitude of the ground state bleach (GSB) at 684 nm from the TA spectrum 7.7 X 10^{-5}.

❖ Thus, triplet concentration could be obtained as 4.15 mM, Amplitude of ESA at 555 nm from the TA spectrum= 3.65 X 10^{-4}

❖ Thus, considering the triplet concentration, triplet extinction coefficient (molar absorptivity) would be 87.95 X 10^3 M^{-1} cm^{-1}.

It is important to note that, as GSB represents the population of photoinduced species, the overall triplet concentration has been considered based on TA amplitude at the GSB signal. While, to avoid any spectral overlap from singlet (even though at 40 ps this possibility is negligible) the molar absorptivity of triplets is calculated from the 555 nm ESA band where triplet states explicitly absorb.

If I_0 and I correspond to incident and transmitted pump light, the fraction of light intensity transmitted (I/I_0) after passing through the sample is a function of initial concentration of S_1 i.e. [S_1]

$$^{I}/_{I_0} = 1 - 10^{-\epsilon(630\ nm)cl} = 0.064$$

[calculated ground state molar absorptivity at 630 nm is 19205.3 M^{-1} cm^{-1}]

Additionally, [S_1] also depends on the total number of photons per pump pulse.

Photon/pulse= 1.46 X 10^9

Now, the number of absorbed photons in the interaction volume will be equal to the initial population of the excited S_1 state. Therefore,

$$[S_1] = \frac{(Photon/Pulse) \times (I/I_0)}{N_A \times V} = 0.078 \times 10^{-3} M$$

and using this concentration the ground state molar absorptivity of singlets at 555 nm can be obtained as ~58.344 X 10^3 M^{-1} cm^{-1}.

Note that in the above equation of [S_1], N_A and V, respectively, represent Avogadro's number and the interaction volume of the pump laser during excitation. The V is calculated as follows.

$$V = \text{Area X h} = (\pi.r^2) \text{ X h} = 0.2 \text{ X } 10^{-7} \text{ cm}^3 = 0.2 \text{ X } 10^{-10} \text{ L}$$

where r and h correspond to the radius of the pump beam (250 μm) and the height of the interaction volume (i.e., the thickness of the film, ~10 nm).

Since, the 555 nm region appears as the signature peak for the triplets, the resultant TA signal amplitude at 555 nm can be represented as[5]

$$(\Delta OD)_{TA@555\ nm} = -(GS)_{\varepsilon@555\ nm} + (\text{triplet ESA})_{\varepsilon@555\ nm} \qquad \text{.......... (1)}$$

Note, as singlet depletion leads to formation of triplet, a negative value of singlet molar absorptivity at 555 nm is considered in the above equation.[5]

If 'y' mole singlet converts into triplet in our case, then equation (1) can be represented as

$$(\Delta OD)_{TA@555\ nm} = \text{Film thickness x } [(-2y \text{ x } 58.344 \text{ x } 10^3) + (2y \text{ x } 87.95 \text{ x } 10^3)]\text{x}$$

$$3.65 \text{ x } 10^{-4} = 10 \text{ x } 10^{-7} \text{ x } 59.22y \text{ x} 10^3$$

y = ~6.16 X 10^{-3} M singlet undergoes singlet fission for the formation of triplets under the experimental conditions.

Triplet yield = 2 x (6.16 x 10^{-3}/0.078 x 10^{-3}) = ~158.0 %

Note that, as singlets that undergo fission to from two triplet states, the final triplet concentration is multiplied by factor 2 and the final value is associated with a standard deviation of ~3-5%, based on our different batch of experiments.

7 NMR

7.1 ^{1}H-NMR Ace-PCSi

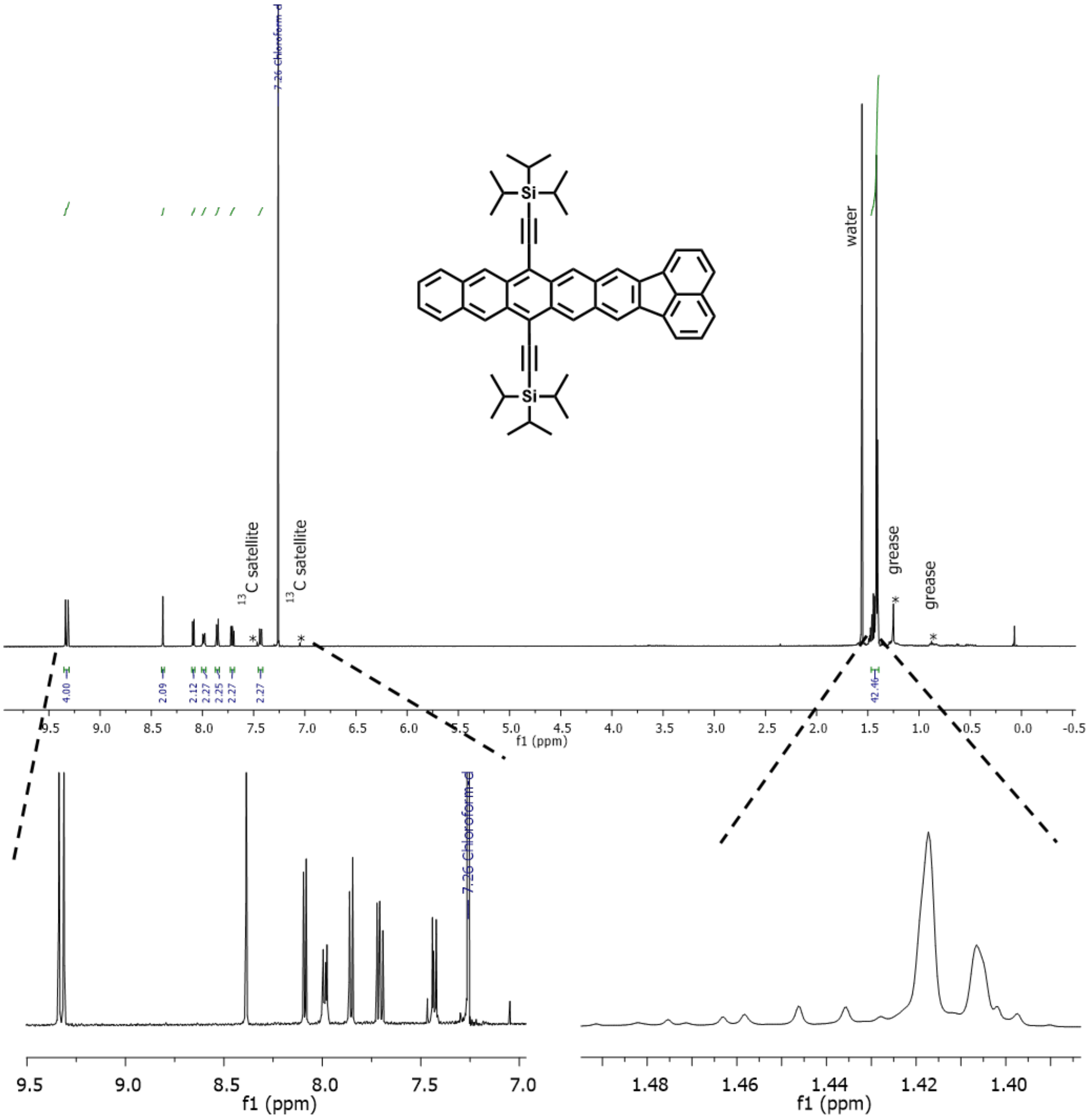

Figure S23. ^{1}H NMR Spectra of **Ace-PCSi** measured in $CDCl_3$ at room temperature.

7.2 ^{13}C-NMR Ace-PCSi

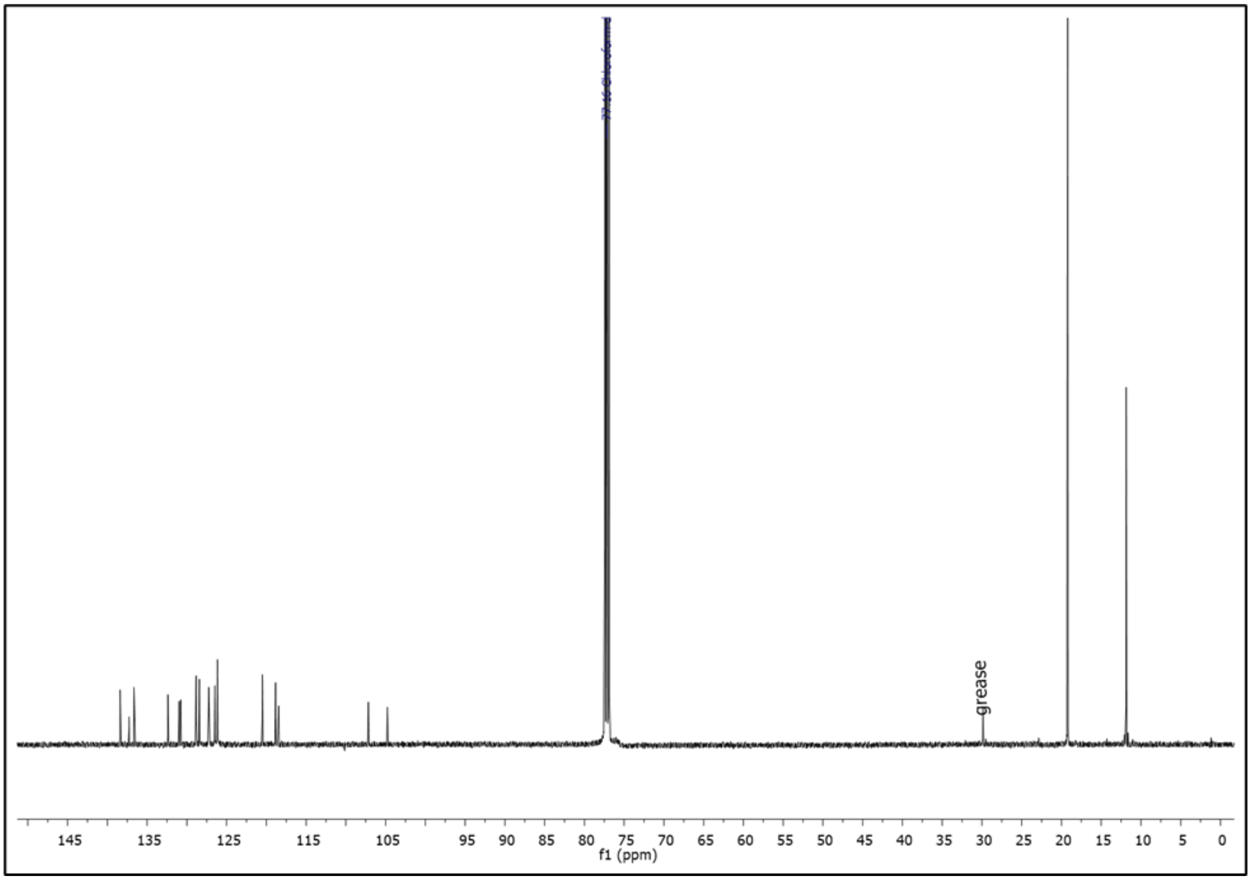

Figure S24. ^{13}C NMR Spectra of **Ace-PCSi** measured in $CDCl_3$ at room temperature.

7.3 ^{1}H-NMR Ace-PCPh

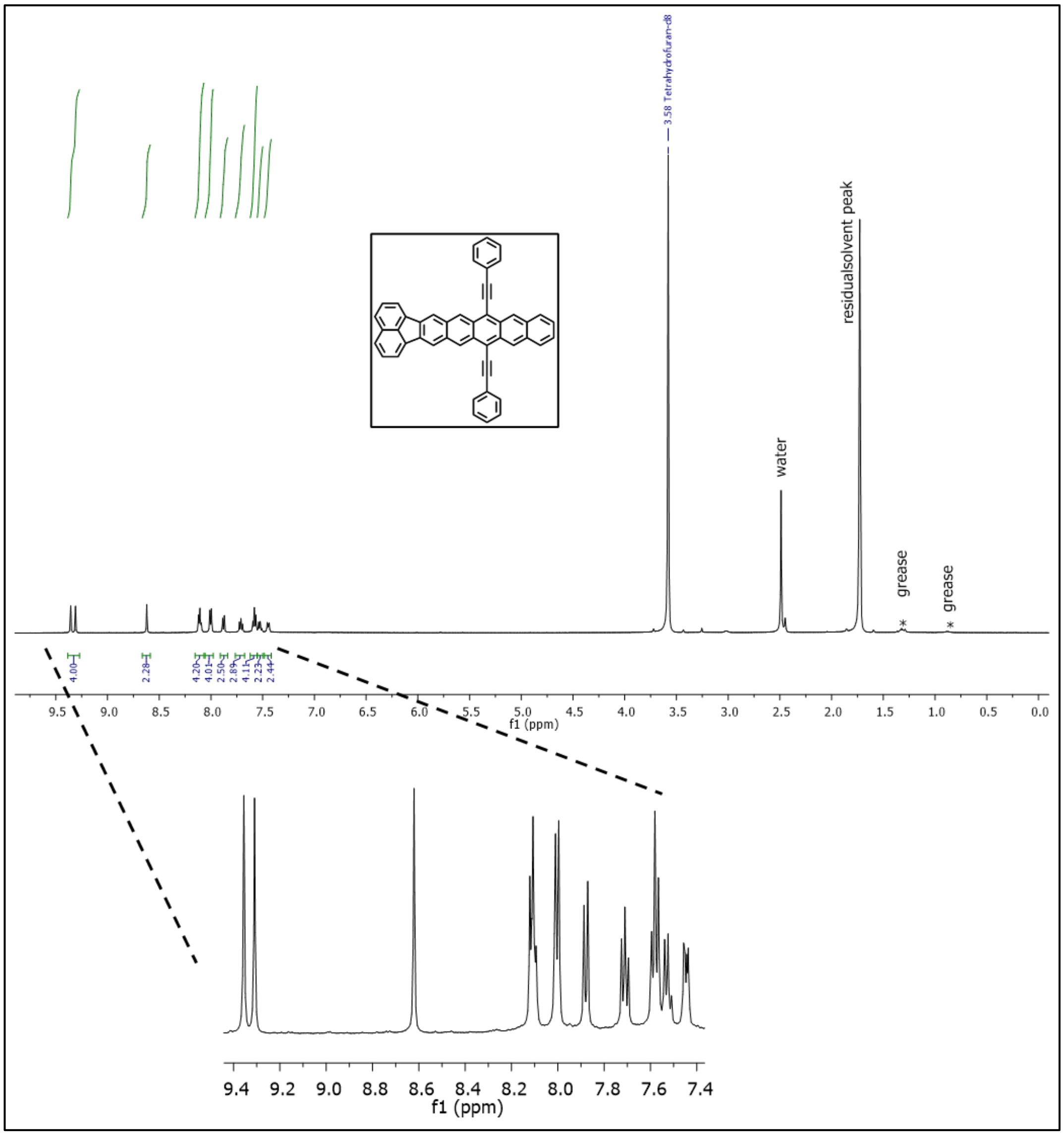

Figure S25. ^{1}H NMR Spectra of **Ace-PCPh** measured in d8-THF at room temperature.

7.4 ^{13}C-NMR Ace-PCPh

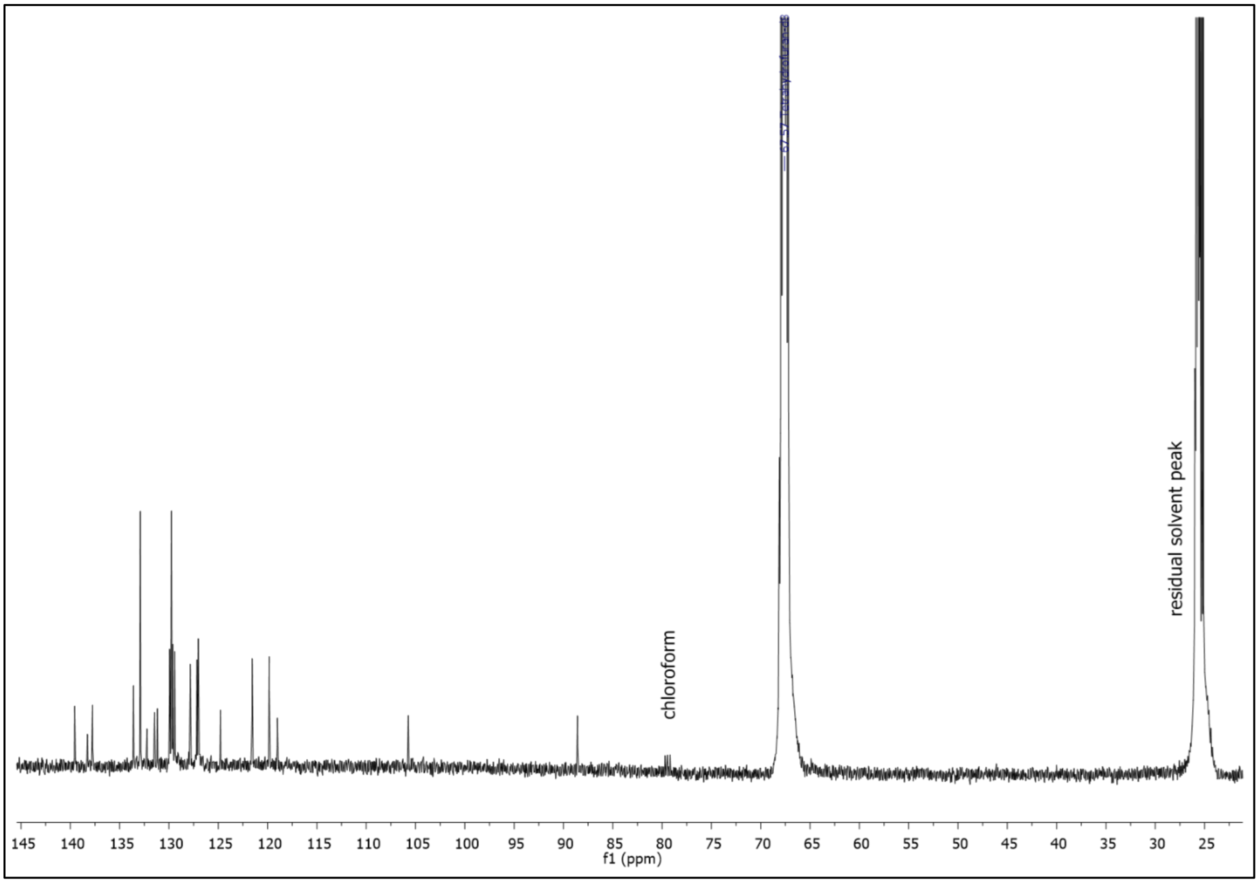

Figure S26. ^{13}C NMR Spectra of **Ace-PCPh** measured in d8-THF at room temperature.

7.5 COSY Ace-PCSi

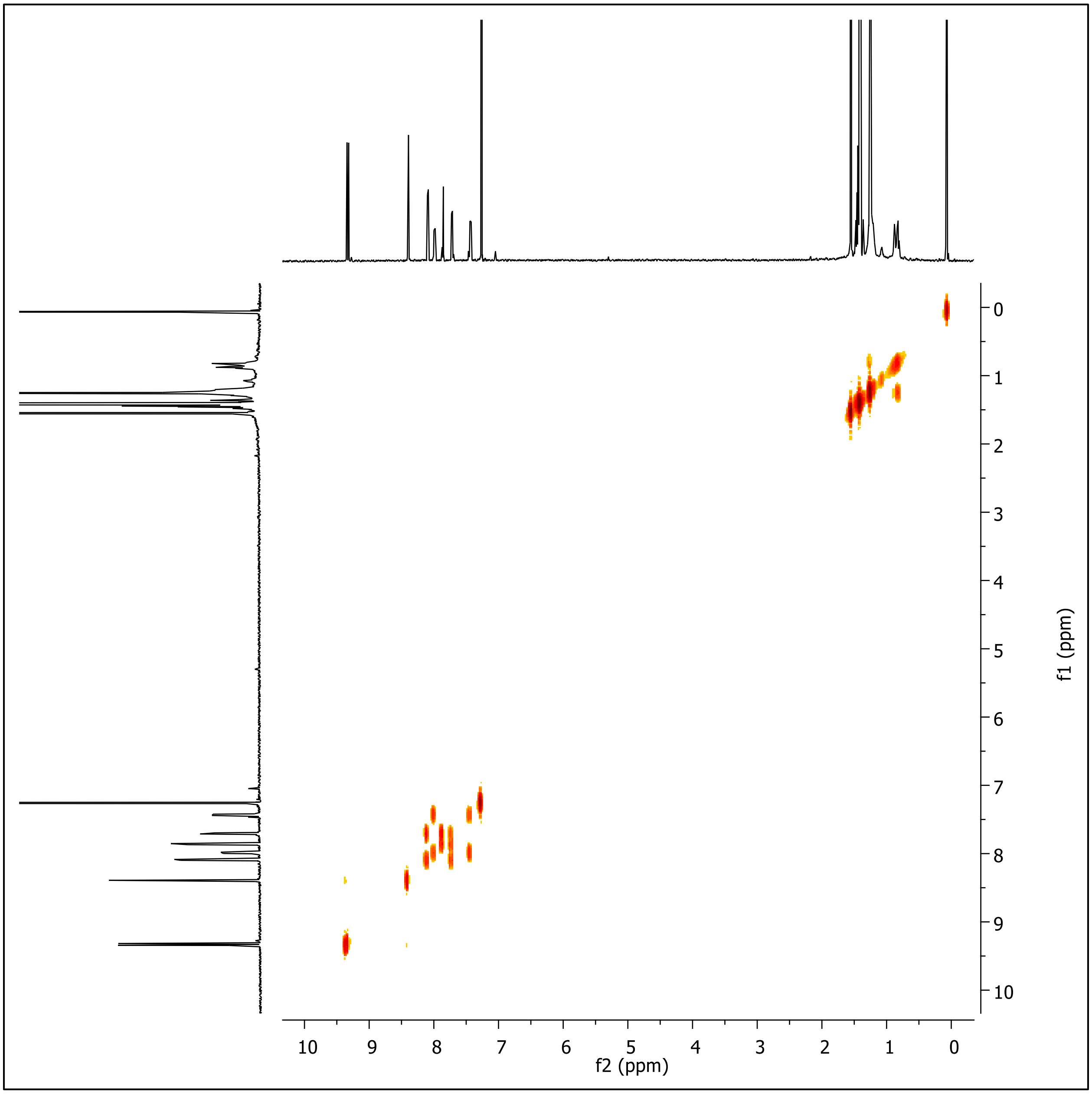

Figure S27. COSY Spectra of **Ace-PCSi** measured in $CDCl_3$ at room temperature.

7.6 ^{1}H-NMR BEP-Si

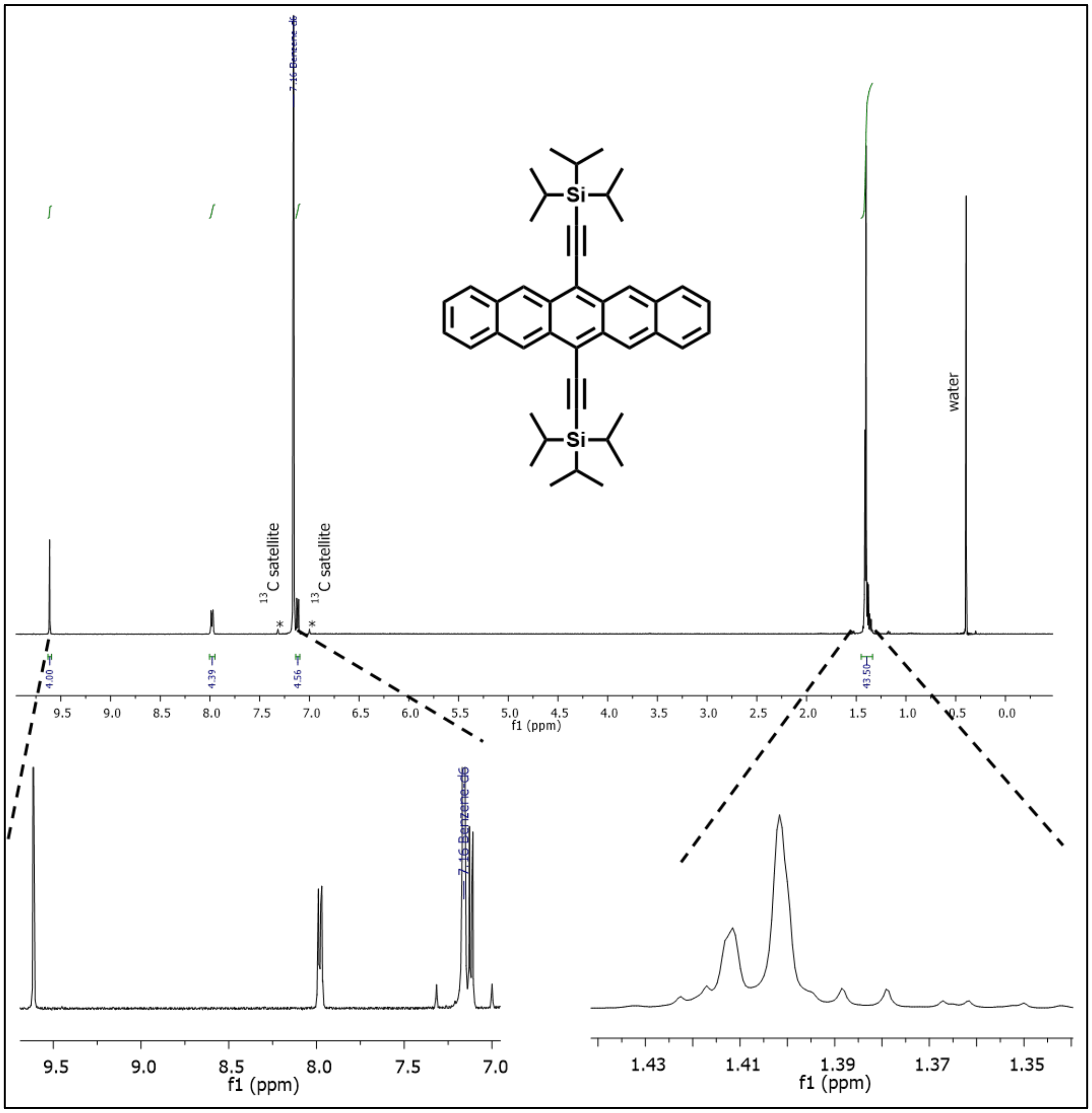

Figure S28. ^{1}H NMR Spectra of **BEP-Si** measured in C_6D_6 at room temperature

7.7 ^{13}C-NMR BEP-Si

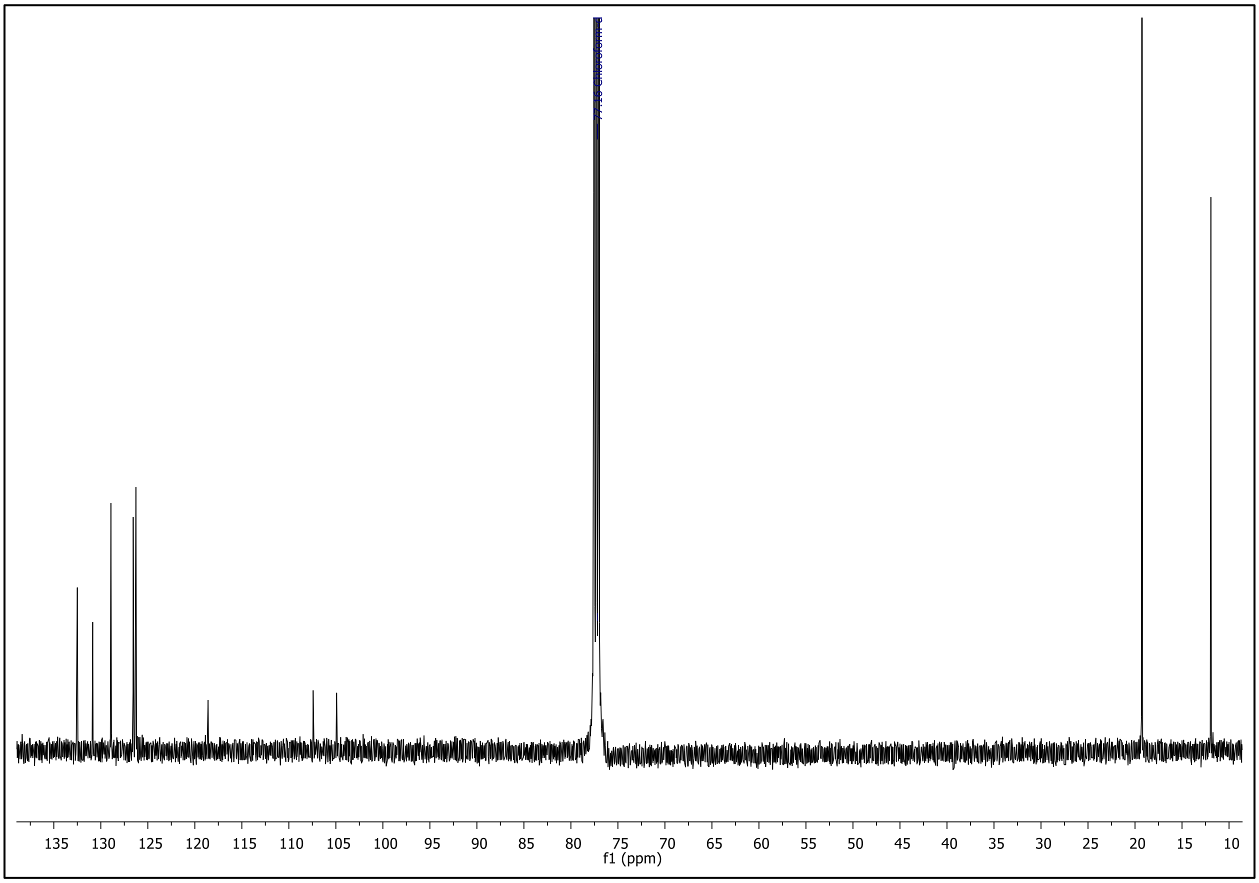

Figure S29. ^{13}C NMR Spectra of **BEP-Si** measured in $CDCl_3$ at room temperature.

7.8 COSY BEP-Si

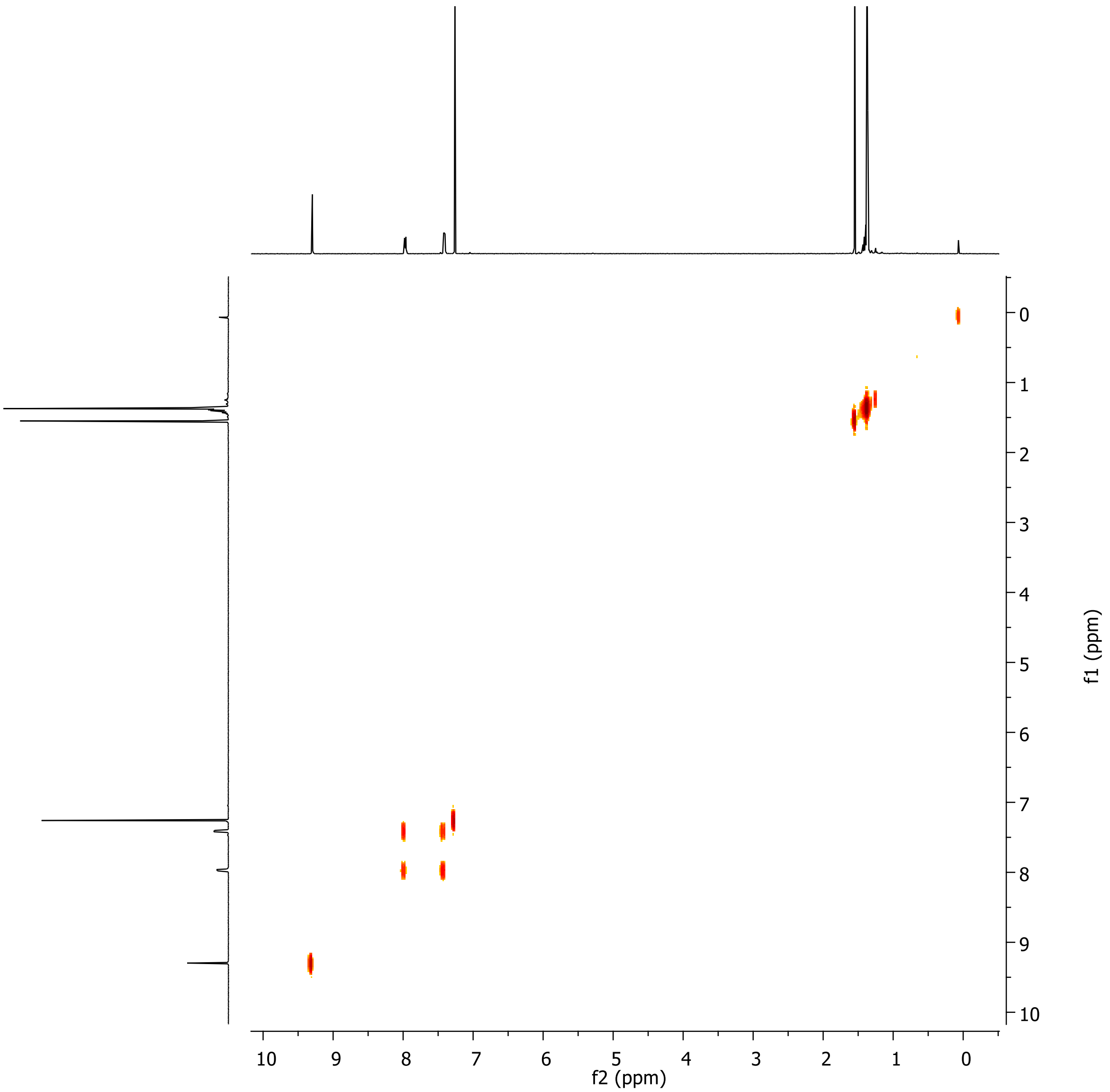

Figure S30. COSY Spectra of **BEP-Si** is measured in $CDCl_3$ at room temperature.

8 Mass- Spectrometry

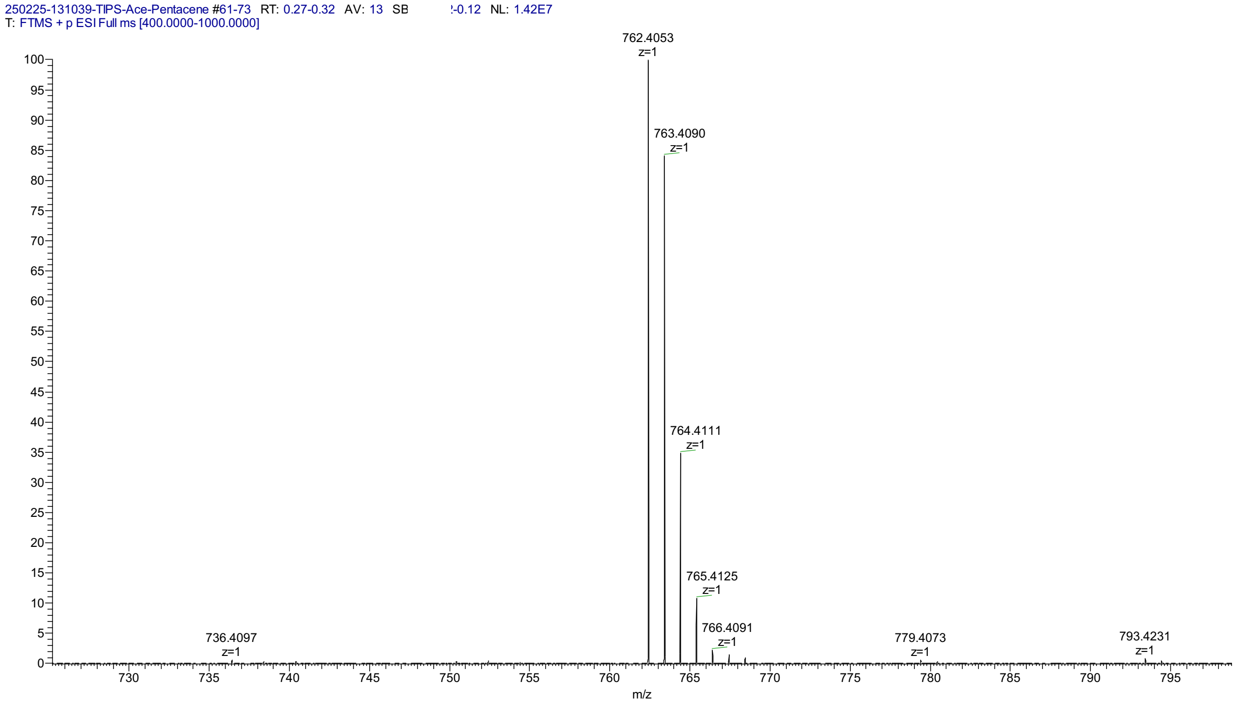

Figure S31. ESI Mass-spectrometry of **Ace-PCSi.**

9 X-Ray Crystallographic Data

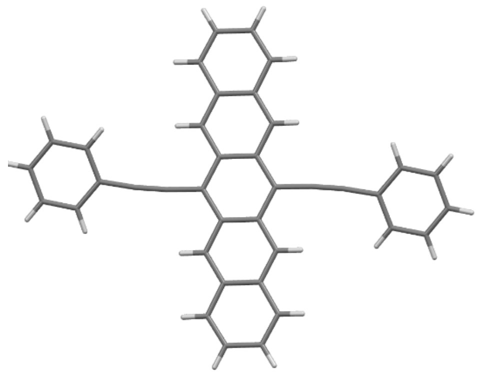

Figure S32. Crystal Structure of **BEPPh**.

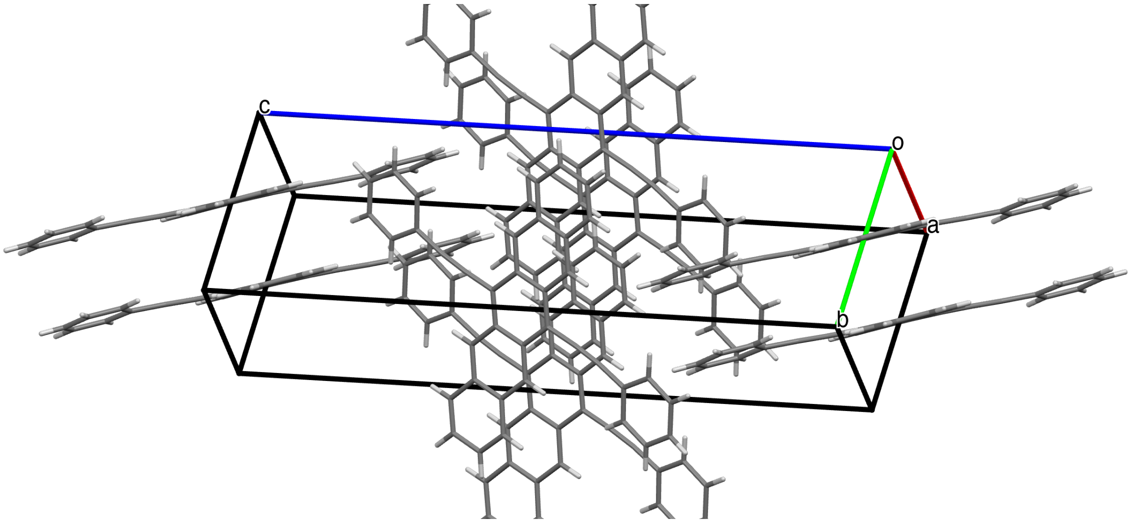

Figure S33. Crystal Packing Motif of **BEPPh**.

a) b)

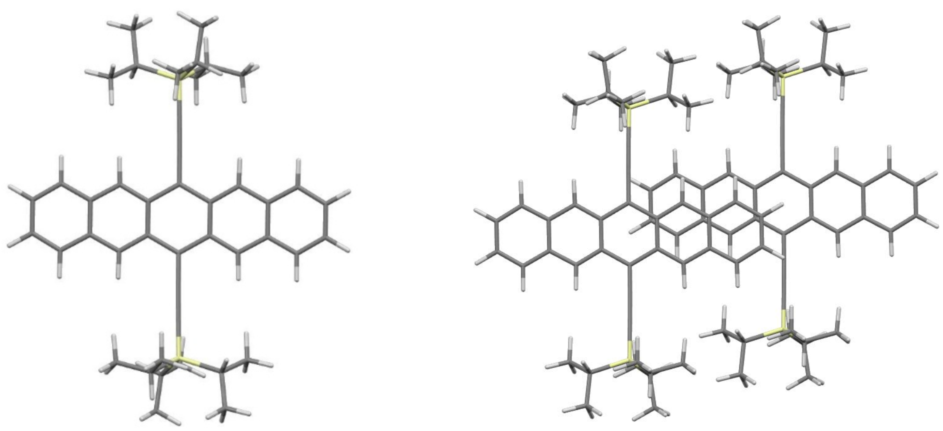

Figure S34. Crystal Structure of **BEP-Si (a)** and its packing molecular overlap **(b) (**CCDC deposition number 172476)**.**

10 Thin-Film-Characterization

10.1 Thin-Film-PXRD

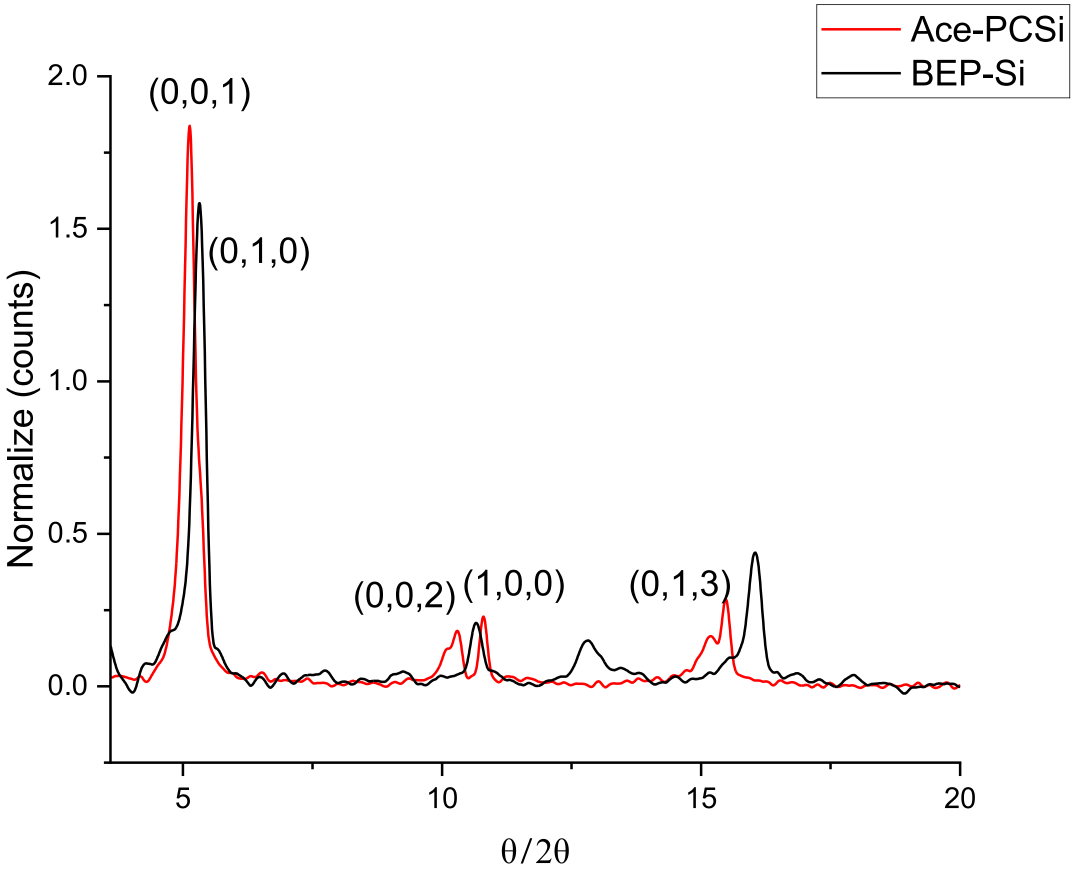

Figure S35. PXRD intensity profile (top) of **Ace-PCSi** (**red**) and **BEP-Si** (**black**) on thin film.

10.2 Thin-Film thickness - AFM

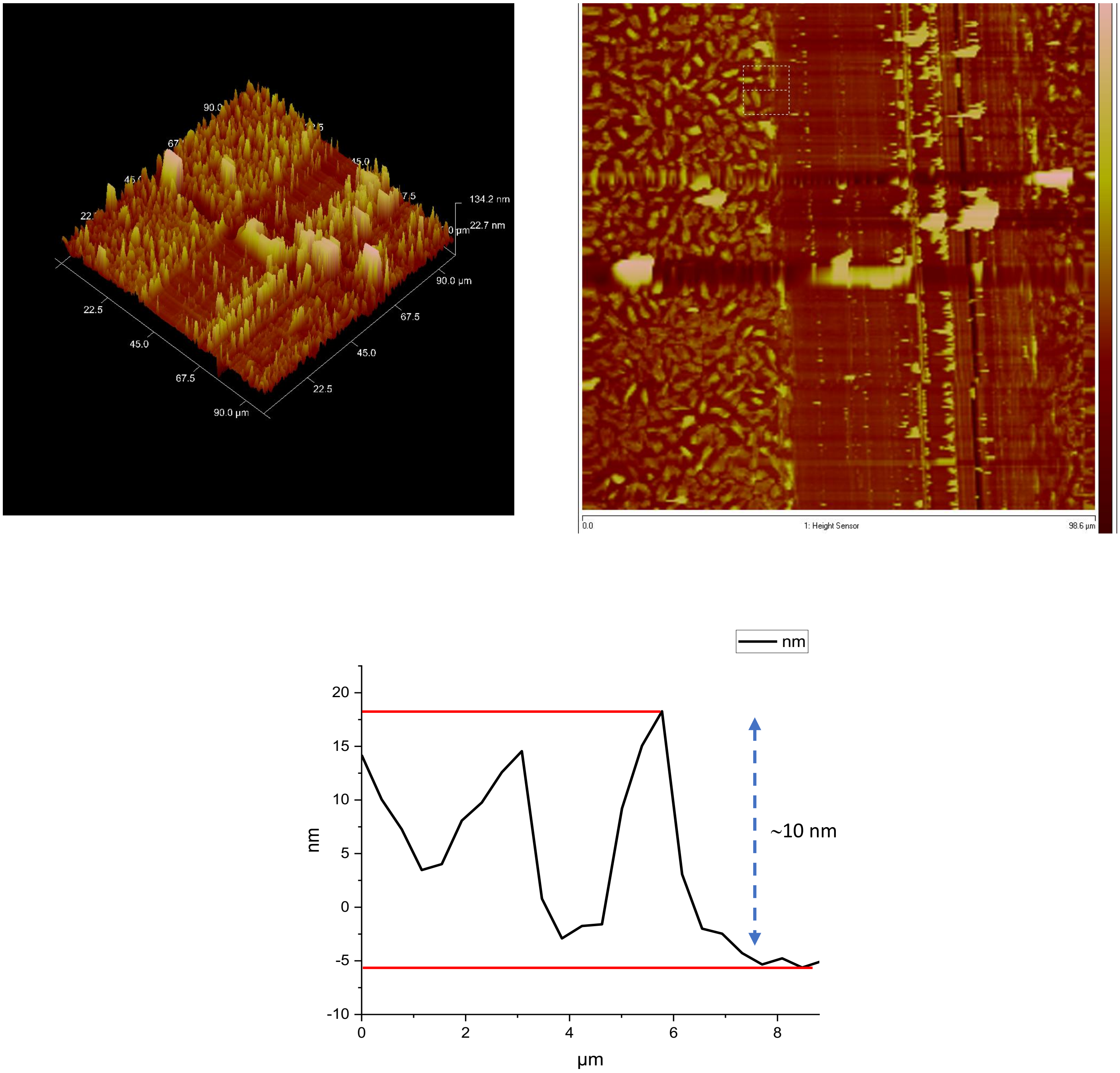

Figure S36. Thickness of a Thin Film **Ace-PCSi** measured using AFM.

10.3 Thin-Film thickness – Ellipsometry

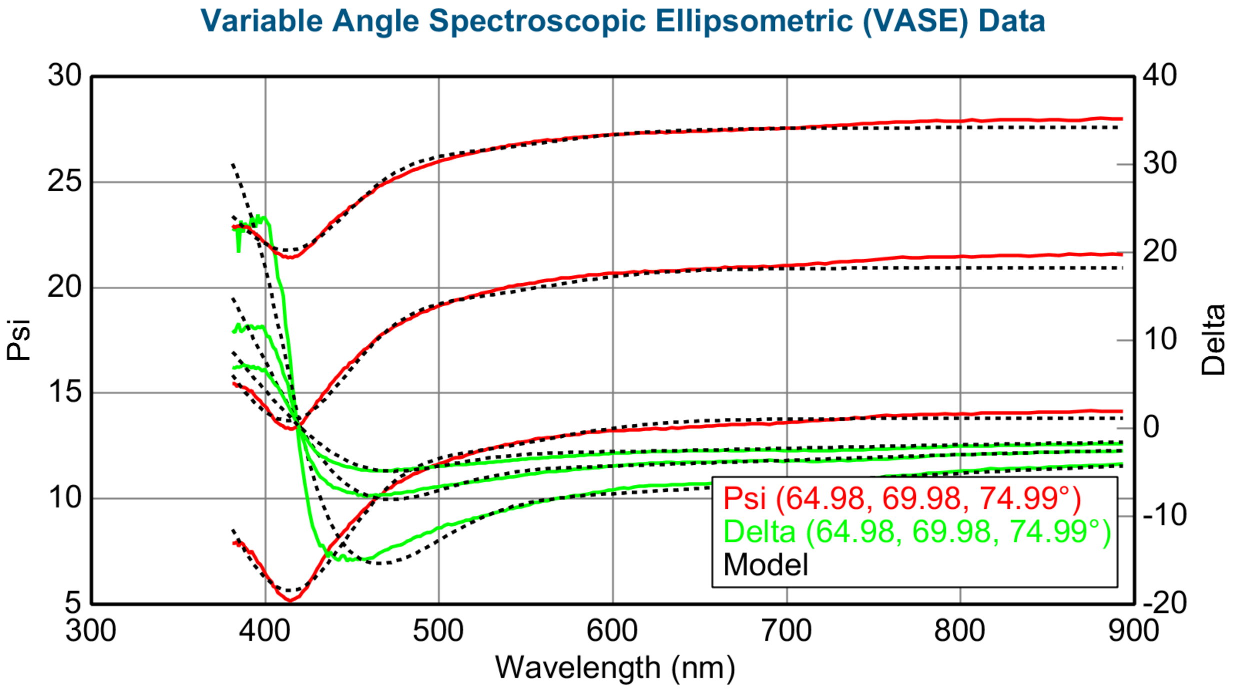

MSE = 7.532

Roughness = 28.53 ± 8.432 nm

Thickness # 1 = 10.39 ± 3.505 nm

n of B-Spline @ 632.8 nm = 2.74576

k of B-Spline @ 632.8 nm = 0.11447

Figure S37. Thickness of a Thin Film **Ace-PCSi** measured using Ellipsometry.

11 List of Acenes and their respective Triplet Yields

Table S3. A summary of triplet yield observed in different analogous acenes exhibiting exothermic singlet fission.

System	Structure	Triplet yield	State	Ref.
Tetracene	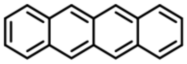	~200%	TF	*Nat. Chem*. 2012, *4*, 840–845.
Phenyl tetracene Nanoparticle<br><br>Phenyl tetracene carboxylic acid Nanoparticle	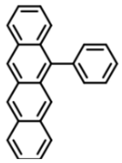<br>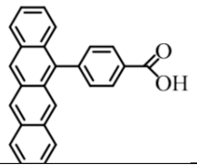	67±15%<br><br>133±10%	Solution	*J. Mater. Chem. C*, 2019, *7*, 11090-11098.
Phenyl tetracene nanoparticles with polyethylene glycol (n=1 and 2)	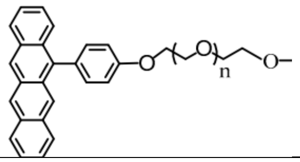	60.6% and 53.3% when n=1 and 2, respectively	Solution	*J. Photochem. Photobiol. A* 2020, *397*, 112597.
Pentacene	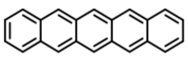	160-180%	TF	*J. Am. Chem. Soc*. 2011, *133*, 11830–11833.
TIPS-Pentacence nanoparticles	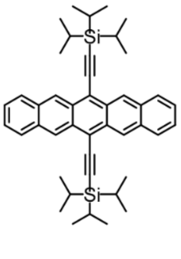	99.9%	Solution	*Chem. Mater*. 2018, *30*, 4409–4421.

TIPS-Pentacence and its heteroatom derivatives	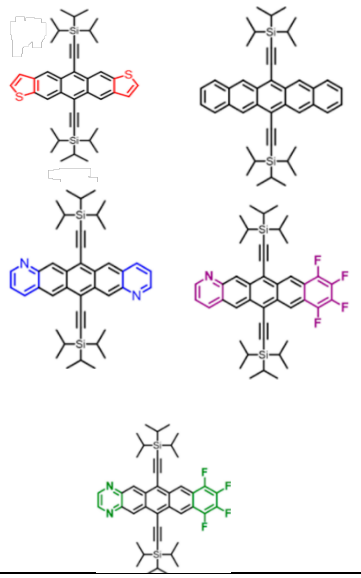	117%, 124%, 140%, 132% and 136%	Solution	*J. Am. Chem. Soc.* 2016, *138*, 6739−6745.
TIPS-Pentacence	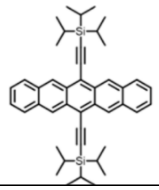	144±25%	TF	*J. Am. Chem. Soc.* 2012, *134*, 386–397.
Diaza- TIPS-Pentacence	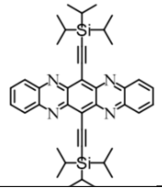	---	TF	*J. Phys. Chem. Lett.* 2014, *5*, 2425–2430.
Acenaphthylene-Fused Pentacene (Ace-PCSi)	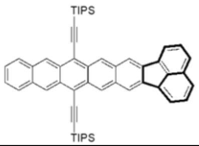	158±5%	TF	This work

12. TREPR Study

Table S4. Parameters for computations of the delay time dependence of the EPR spectra considering the conformation changes between TT_1 and TT_2 states by the exciton diffusion accompanying the ultimate T+T dissociation.[6,7]

		J /MHz	D /MHz [a]	E /MHz [a]	D_{SS} / MHz	Euler angles [b] / degrees (α, β, γ)	Dipolar angles [c]/ degrees	$\bar{\nu}_{vib}$/ cm^{-1}	ΔE_{12} / cm^{-1} [d]	k_{Diss} [e]/ s^{-1} k_{Back} [e]/ s^{-1}	k_{Rec} [e]/ s^{-1}
80 K	TT_1	-2.9×10^5	1,050	15	-95	$\alpha = 90$ $\beta = 180$ $\gamma = -90$	$\theta_2 = 20$ $\phi_2 = 40$	0.24	70	1.6×10^7	3.0×10^6
	TT_2	-1.1×10^4	1,050	15	-95	$\alpha = 90$ $\beta = -76$ $\gamma = -30$	$\theta_2 = 20$ $\phi_2 = 40$				-
	T+T	0	1,050	15	0	$\alpha = 90$ $\beta = 100$ $\gamma = -90$	$\theta_2 = 30$ $\phi_2 = 90$	-	-	4×10^5	-

[a] Zero-field splitting parameters in $\boldsymbol{H}_{zfs} = D\{S_z^2 - S(S+1)/3\} + E\left(S_x^2 - S_y^2\right)$ for each **Ace-PCSi** triplet in the $T_A T_B$ multiexciton.

[b] Conformation of the principal axes in $\boldsymbol{H}_{zfs}$ of the T_B component with respect to the principal axes of T_A component in the $T_A T_B$ multiexciton with the x-convention.

[c] Direction for the T_B component was set by the polar angles (θ_2 and ϕ_2) with respect the (X_1, Y_1, Z_1) principal axes in T_A.

[d] The energy gap between TT_1 and TT_2 states represented by $\Delta E_{12} = E(TT_2) - E(TT_1)$.

[e] k_{Diss} and k_{Back} are dissociation ($^5TT_1$ -> T+T) and reversing (T+T -> $^5TT_1$) rate constants, respectively.

13. References

1. T. Lu and F. Chen, *Journal of Computational Chemistry*, 2012, **33**, 580–592.
2. http://glotaran.org/ (accessed in 2024).
3. J. J. Snellenburg, S. Laptenok, R. Seger, K. M. Mullen, I. H. M. Stokkum, *J. Stat. Software* **2012**, *49*, 1−22
4. J. E. Anthony, J. S. Brooks, D. L. Eaton, S. R. Parkin, *J. Am. Chem. Soc.* **2001**, *123*, 9482-9483.
5. A. Nandi, B. Manna, R. Ghosh, *J. Phys. Chem. C* **2021**, *125*, 2583–2591.
6. Y. Kobori, M. Fuki, S. Nakamura, T. Hasobe, *J. Phys. Chem. B* **2020**, 124, 9411-9419.
7. S. Nakamura, H. Sakai, H. Nagashima, M. Fuki, K. Onishi, R. Khan, Y. Kobori, N. V. Tkachenko, T. Hasobe, *J. Phys. Chem. C* **2021**, 125, 18287-18296.

14. Author Contribution

H. W. and J. A. designed the project. J. A. and S. S. conducted the synthesis and characterization. S. S and A. D carried out initial spectral studies of the compounds. S. D. and F. D. designed and

carried out the experiments for transient absorption spectroscopy and data analysis. S. T. and Y. K. designed and carried out the experiments for time-resolved electron paramagnetic resonance spectroscopy. S. S performed all the computations. V. N. N. collected the X-ray diffraction data and solved the crystal structures. S. S., T. P., and S. L. designed and conducted film preparation and measurements. All authors contributed to data analysis and manuscript writing
